# Fluorination Effect on Lithium- and Manganese-Rich Layered Oxide Cathodes

**DOI:** 10.1021/acsenergylett.3c02697

**Published:** 2024-02-27

**Authors:** Faxing Wang, Peng Zuo, Zhichen Xue, Yijin Liu, Chongmin Wang, Guoying Chen

**Affiliations:** † Energy Storage and Distributed Resources Division, Lawrence Berkeley National Laboratory, Berkeley, California 94720, United States; ‡ Environmental Molecular Sciences Laboratory, Pacific Northwest National Laboratory, Richland, Washington 99354, United States; § Stanford Synchrotron Radiation Lightsource, SLAC National Accelerator Laboratory, Menlo Park, California 94025, United States

## Abstract

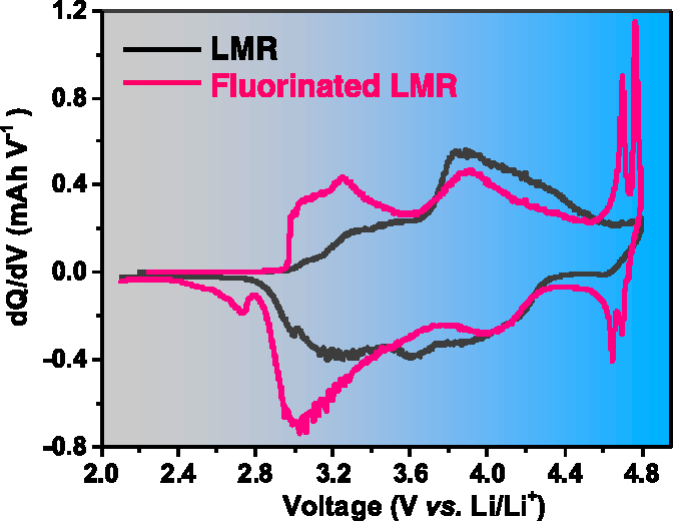

Lithium- and manganese-rich (LMR) layered oxides are promising high-energy cathodes for next-generation lithium-ion batteries, yet their commercialization has been hindered by a number of performance issues. While fluorination has been explored as a mitigating approach, results from polycrystalline-particle-based studies are inconsistent and the mechanism for improvement in some reports remains unclear. In the present study, we develop an *in situ* fluorination method that leads to fluorinated LMR with no apparent impurities. Using well-defined single-crystal Li_1.2_Ni_0.2_Mn_0.6_O_2_ (LNMO) as a platform, we show that a high fluorination level leads to decreased oxygen activities, reduced side reactions at high voltages, and a broadly improved cathode performance. Detailed characterization reveals a particle-level Mn^3+^ concentration gradient from the surface to the bulk of fluorinated-LNMO crystals, ascribed to the formation of a Ni-rich Li_*z*_Ni_*x*_Mn_2–*x*_O_4–*y*_F_*y*_ (*x* > 0.5) spinel phase on the surface and a “spinel-layered” coherent structure in the bulk where domains of a LiNi_0.5_Mn_1.5_O_4_ high-voltage spinel phase are integrated into the native layered framework. This work provides fundamental understanding of the fluorination effect on LMR and key insights for future development of high-energy Mn-based cathodes with an intergrown/composite crystal structure.

Lithium- and manganese-rich (LMR) layered oxides, discovered more than two decades ago, have the potential to replace the LiNi_1–*x*–*y*_Mn_*x*_Co_*y*_O_2_ (NMC)-type cathodes currently used in commercial lithium-ion batteries (LIBs). These materials offer a higher specific capacity (∼250 mAh·g^–1^), higher energy density (>800 Wh·g^–1^), better thermal stability, and lower cost.^1^ Several grand challenges, however, have hindered their commercial adoption, particularly impedance rise at low state of charge, capacity, and voltage fade with cycling. While the combined cationic and anionic redox activities contribute to the large capacity of LMR cathodes, cycling is often accompanied by irreversible loss of oxygen from the lattice.^2−4^ The removal of both Li and lattice oxygen upon charging to high voltages (e.g., 4.8 V) results in the migration of the transition metals (TMs) from the undercoordinated to fully coordinated octahedral sites in the Li layer. Such irreversible TM migration and Li/O loss lead to structural changes from the layered to a LiMn_2_O_4_-type spinel phase and, consequently, voltage decay, capacity fade, poor initial Coulombic efficiency (ICE), and sluggish reaction kinetics.^5−8^

To address these performance issues, cation doping (e.g., Na, Mg, Cr, etc.), anion doping (e.g., PO_4_, SO_4_, etc.), surface coating (e.g., Al_2_O_3_, spinel phase, etc.), compositional engineering, and morphological modifications have all been explored in the past.^7,9−13^ Since O loss often occurs on particle surfaces, surface coating and/or doping of the O sublattice have shown great promise. Among them, fluorination is widely explored.^14−24^ Due to the lower highest occupied molecular orbital (HOMO) level of F^–^ anions than that of the O^2–^ anions, F doping enables stronger metal–fluorine (M–F) bonds as compared to the M–O bands. F doping can also protect the electrode surface against HF attack, a common issue associated with the LiPF_6_-based electrolytes.^24^ Furthermore, the lower negative charge in F reduces the average cation oxidation state in the oxide, leading to increased capacity contribution from the TM redox couples.^25^

In theory, F doping in the classic NMC-type phases is considered unfavorable due to the low solubility of LiF in a well-ordered layered crystal structure. First-principles calculations showed that the ability to incorporate fluorine into lithium-excess transition-metal oxides is closely related to their cation disordering degree.^23^ Disorder in the cation sublattice can create local Li-rich environments, thus, increasing LiF solubility. Experimentally, several groups reported that fluorine is present as a LiF coating on the surface instead of a dopant in the layered LMR lattice.^20,21^ This was largely supported by ^19^F magic-angle spinning nuclear magnetic resonance (NMR) studies. A few investigations also showed that after fluorination, a high-voltage (HV) LiNi_0.5_Mn_1.5_O_4_ spinel layer can form on an LMR surface.^19,22^ However, it is unclear what role F plays or even where it is located in these structures. Since the layered LMR and the HV-spinel structures share the same cubic closed oxygen sublattice that enables their integration at the atomic level, a series of “layered–layered-spinel” oxide samples with the high-capacity 0.5Li_2_MnO_3_·0.5LiMn_0.5_Ni_0.5_O_2_ (“layered–layered”) and HV LiNi_0.5_Mn_1.5_O_4_ (spinel) components were also designed and prepared by Thackeray et al. without the involvement of fluorination.^26−28^ In their studies, the excess Li^+^ cations accompanying the irreversible O_2_ release were reaccommodated in the HV spinel to form the overlithiated Li_2_Ni_0.5_Mn_1.5_O_4_ phase upon discharging to below 3 V, thereby increasing the ICE and discharge capacity. They further proposed that integrating HV spinel structure into layered LMR may alleviate Jahn–Teller distortion and Mn dissolution, owing to the improved tolerance of HV spinel component with an average Mn oxidation state of 3.33+ instead of 3+ at the fully lithiated state.^27,28^ Furthermore, particle surface layer construction was also used to tune the chemical environment for redox-active oxygen and reduce the extent of surface lattice oxygen escape. The authors attributed the decreased irreversible oxygen loss to the formation of Ni-enriched spinel layers on the surface.^19,29^

While the benefits of LMR fluorination have been reported,^14−19^ the underlying mechanism as well as fluorine solubility remain unclear. A number of factors such as phase transition, side reactions, and polarization are known to play a role in the properties and electrochemical performance of LMR cathodes.^30−32^ As most studies used polycrystalline-based aggregated secondary particles, the presence of grain boundaries and particle-level porosity adds variability and complexity, particularly in understanding the nature of surface F and its distribution. In this work, we use a modified molten-salt technique to synthesize fluorinated and unfluorinated Li_1.2_Ni_0.2_Mn_0.6_O_2_ (LNMO) single-crystal samples. Through detailed characterization using synchrotron X-ray diffraction (XRD) and soft X-ray absorption (sXAS) spectroscopy, X-ray photoelectron spectroscopy (XPS), scanning transmission electron microscopy (STEM) - electron energy loss spectroscopy (EELS), and energy-dispersive X-ray spectroscopy (EDX), we show that high fluorination levels promote a gradient distribution of Mn^3+^ concentration from the surface-to-bulk of the particle as well as the presence of a spinel phase in both surface and bulk. While the surface spinel appears to be Ni-enriched Li_*z*_Ni_*x*_Mn_2–*x*_O_4–*y*_F_*y*_ (*x* > 0.5), the bulk has a “spinel-layered” coherent structure where the LiNi_0.5_Mn_1.5_O_4_ HV-spinel phase is integrated into the native layered framework. Electrochemical studies further confirm the improved performance in the fluorinated-LNMO (F-LNMO) cathodes, including enhanced specific capacity and energy density as well as better retention of capacity, voltage, and energy density.

## Synthesis and Properties of F-LNMO Single Crystals

. The common fluorination methods involve solid-state mixing of LiF and preformed LMR particles to obtain fluorinated-LMR, known as the postsynthesis fluorination technique. Due to the high thermodynamic stability of LiF and the stronger affinity of F anions toward Li than the TMs, this approach often leads to the formation of LiF secondary phase on the surface of LMR particles rather than the incorporation of F anions into the oxygen anion sublattice.^15,21^ Here we investigate an alternative *in situ* fluorination method based on a molten-salt synthesis technique. A series of LNMO and F-LNMO single crystals with the general formula of Li_1.2_Ni_0.2_Mn_0.6_O_2–*x*_F_*x*_ (*x* = 0, 0.01, 0.025, and 0.05) were prepared using a modified procedure used in the previous reports.^33,34^ Assuming full incorporation of F into the O lattice and charge compensation achieved through Mn, the target compositions of the samples are Li_1.2_Ni_0.2_Mn^4+^_0.6_O_2_, Li_1.2_Ni_0.2_Mn^4+^_0.59_Mn^3+^_0.01_O_1.99_F_0.01_ (LNMO-F1), Li_1.2_Ni_0.2_Mn^4+^_0.575_Mn^3+^_0.025_O_1.975_F_0.025_ (LNMO-F2.5), and Li_1.2_Ni_0.2_Mn^4+^_0.55_Mn^3+^_0.05_O_1.95_F_0.05_ (LNMO-F5). The corresponding Mn^3+^ contents are 0, 1.6%, 4.2%, and 8.3%, respectively. To synthesize the samples, various fluoride salts were selected and mixed together with the stoichiometric amount of Li/Mn/Ni salt precursors in a KCl flux (mp = 770 °C).^15−19^ Initially, LiF was used as the fluorine source. It was found that upon increasing the F concentration in Li_1.2_Ni_0.2_Mn_0.6_O_2–*x*_F_*x*_ from *x* = 0.01 to 0.1 (LNMO-F10), the LiF impurity content increases continuously, as shown in the XRD patterns in Figure S1a. This indicates that F anions were not well-incorporated into the layered crystal structure.

As the chemical nature of F salts is known to play a critical role in fluorination, the effect of fluoroacidity on the phase purity of the synthesized compounds was then investigated. Much like the pH values used for the proton acidity, fluoroacidity measures the F affinity, with the salts in the basic form being F^–^ givers and those in the acidic form being F^–^ acceptors.^35^ Fluoroacidity is often determined by measuring the concentration of electroactive gas species (SiF_4_), formed between the Si additive and the free F^–^ content in the salt medium using the electrochemical techniques of cyclic and square wave voltammetry.^35,36^Table S1 lists the F-containing salts investigated in this study. The fluoroacidity follows the following order: LiF–KF (51:49) < NaF–MgF_2_ (78:22) < NaF–CaF_2_ (69:31) < LiF–NaF (60:40) < LiF < LiF–CaF (80:20). All ratios in the mixtures are mole ratios. The effect of fluoroacidity on phase purity of the as-synthesized sample is significant, which is clearly demonstrated on the XRD patterns collected on LNMO-F2.5 made with various salts (Figure S1b). The results show that sample phase purity and the fluoroacidity of the F-salt used follow the opposite directions, with the least acidic LiF-KF producing the LNMO-F2.5 phase with the highest phase purity (absence of detectable LiF impurity in XRD). Decreasing the fluoroacidity therefore was found to improve the efficacy of fluorination.

**Figure 1:**
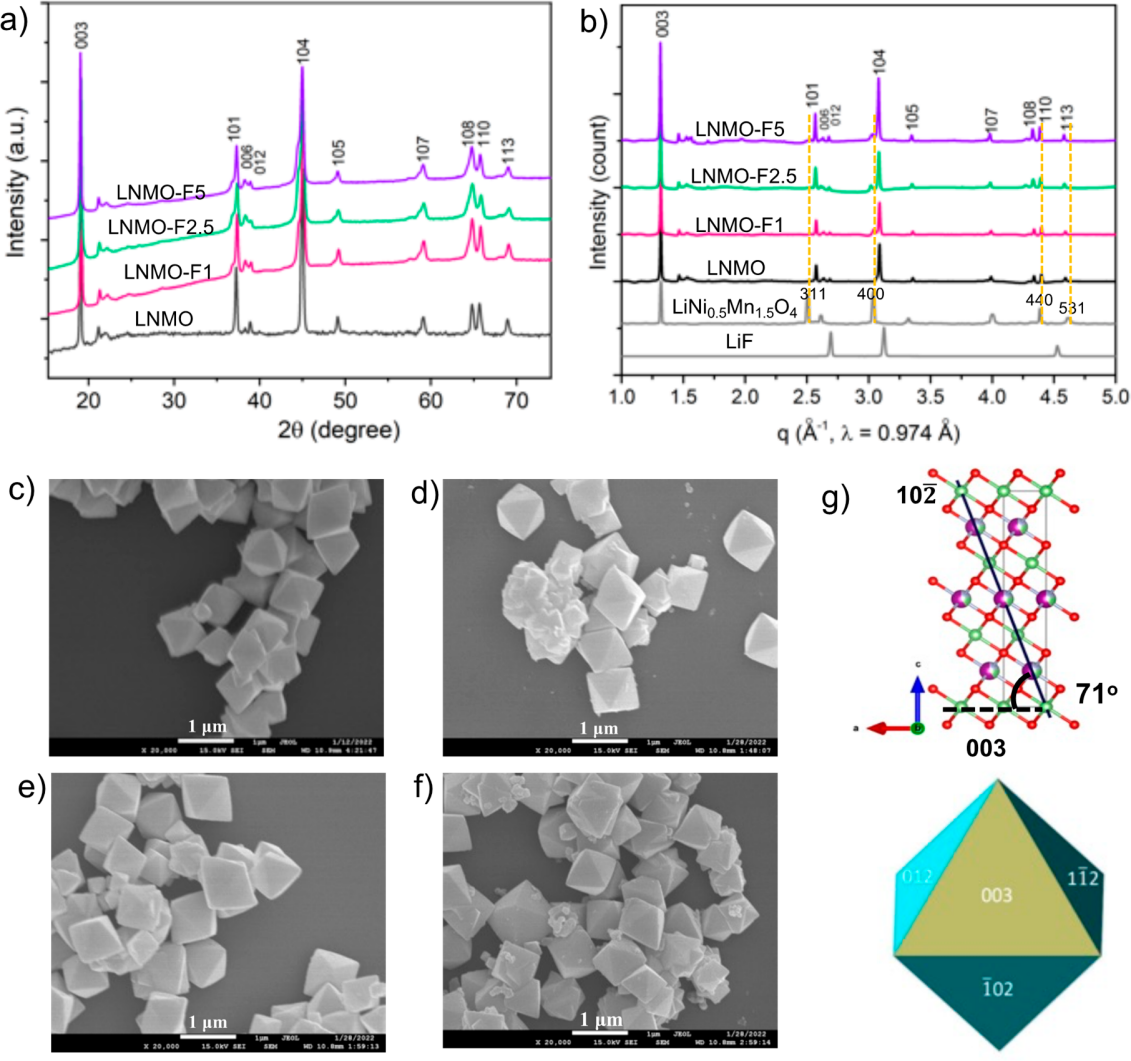
(a) Laboratory and (b) synchrotron powder XRD patterns of as-synthesized crystal samples. XRD patterns of HV spinel LiNi_0.5_Mn_1.5_O_4_ and LiF are added in (b) for reference. SEM images of (c) LNMO, (d) LNMO-F1, (e) LNMO-F2.5, and (f) LNMO-F5. (g) Atomic structure of layered LNMO (top) and a schematic showing the indexed crystal facets (bottom).

Based on these results, LNMO-F1, LNMO-F2.5, and LNMO-F5 samples were synthesized using the *in situ* fluorination method with LiF-KF as the F source. Figure 1a shows the laboratory XRD patterns collected on the LNMO and F-LNMO series. Except the additional peaks between 21° to 23°, the main patterns can be indexed to the layered hexagonal α-NaFeO_2_ structure (space group *R*3̅*m*), which is consistent with previous reports.^37,38^ The clear splitting of the (006)/(012) and (108)/(110) doublets indicates well-ordered layered structure in the hexagonal lattice. Compared to those in LNMO, the (101) and (104) diffraction peaks appear broader in all F-LNMO samples (Figure S1c), suggesting possible presence of secondary phase(s). To this end, synchrotron XRD analysis was used to further investigate the phase purity. Figure 1b shows the data collected on the series of samples along with the two reference samples, LiNi_0.5_Mn_1.5_O_4_ spinel phase (*Fd*3̅*m*) and LiF. The phase purity of LNMO was confirmed. The additional diffraction peaks near the (101) and (104) main peaks in F-LNMO can be indexed to the (311) and (400) peaks of the spinel phase.^39,40^ The intensity of these peaks increases with the F level, with the highest intensity observed on the LNMO-F5 sample. LiF peaks were not detected in all of the samples. Although this does not exclude the possible presence of a very small amount of LiF or amorphous LiF in the sample, the results suggest that the use of more fluorobasic F precursors can be an effective approach to LMR fluorination. The chemical compositions of the samples were further confirmed by inductively coupled plasma optical emission spectroscopy (ICP-OES) measurements. Table S2 shows the metal contents in each sample, which is consistent with all target values. Within the error bar range, the measured F content confirms the presence of F and the increasing F content in the LNMO-F1, LNMO-F2.5, and LNMO-F5 series.

Figure 1c–f shows the scanning electron microscopy (SEM) images of as-synthesized LNMO and F-LNMO samples. All particles show the same single-crystal morphology, with a uniform octahedron shape and an average particle size of ∼1 μm. Fluorination has a negligible effect on both the particle size and morphology. Crystalline orientations of the surface facets were determined by using spherical aberration-corrected STEM imaging combined with focused ion beam (FIB) lithography. When examined along the [010] direction of the layered structure, the terminating planes on the particles are the (003) and (102) planes. The measured angle between them is ∼73°, which is close to the theoretical value of 71° (Figure 1g). Based on the octahedron morphology and the symmetry element 3̅ being perpendicular to the (003) plane, the dominating surface facets in all samples were determined to be (102)-family planes (∼88% or 7 out of 8 facets), with the (003) plane accounting for the rest of the surface (∼12%, or 1 out of 8 facets).

**Figure 2:**
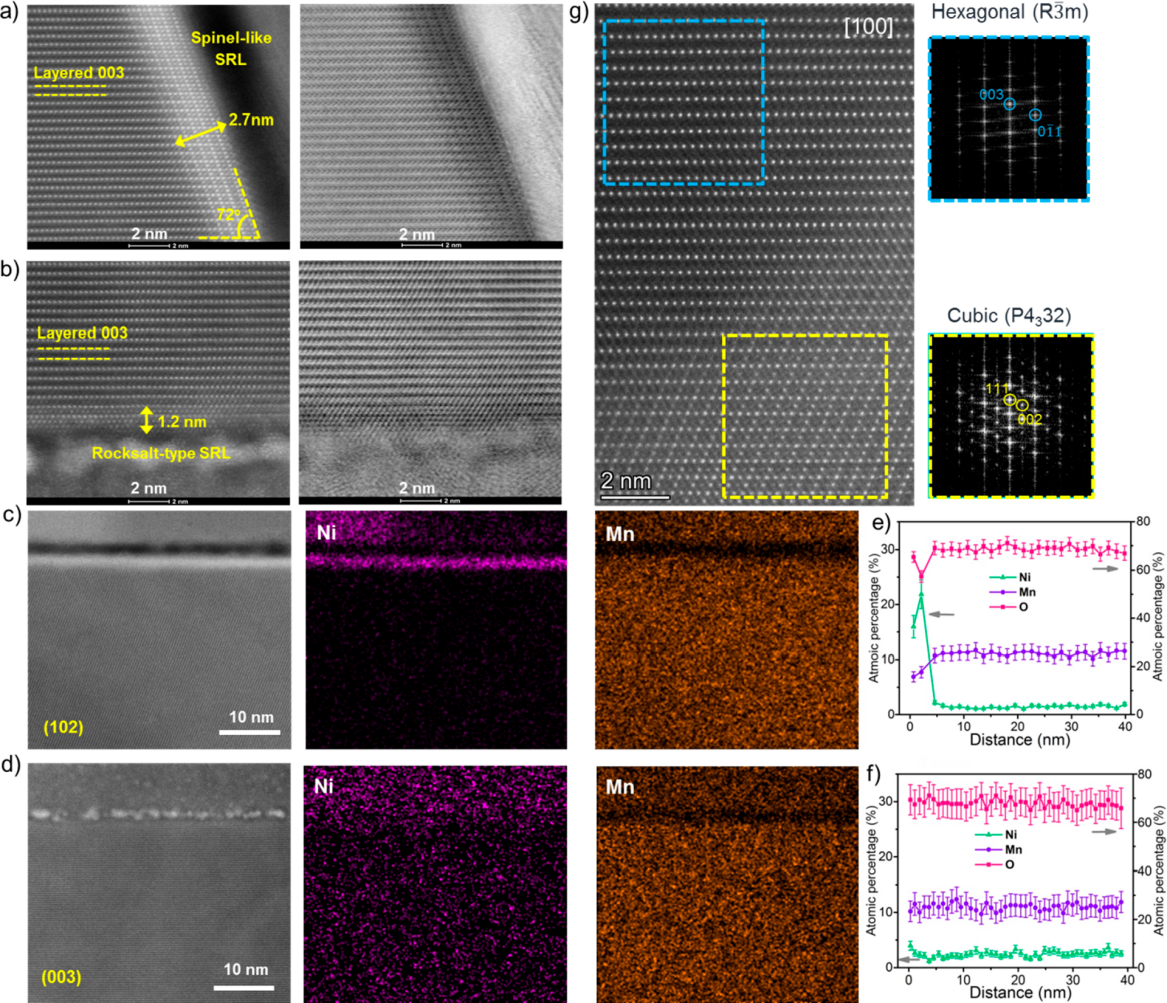
STEM-HAADF (left) and STEM-ABF (right) images of LNMO-F5 along (a) [010] and (b) [101̅] zone axes. (c–f) STEM-EDX mapping and the corresponding estimated atomic percentage at (102) (c, e) and (003) (d, f) crystal planes of LNMO-F5. The probe depth is 40 nm. (g) STEM-HAADF and the corresponding FFT images collected in the bulk region of LNMO-F5.

Further analysis of high angle annular dark field (HAADF) and annular bright field (ABF) STEM images collected along the [101] zone axis of LNMO-F5 crystals shows the presence of a spinel-like surface reconstruction layer (SRL) with a thickness of ∼2.7 nm on the (102) facet (Figure 2a left and right, respectively). The spinel and the layered structures appear crystallographically coherent without the presence of phase boundaries. On the other hand, a rocksalt-type layer with a thickness of ∼1.2 nm was observed on the (003) surface facet, as shown in the HAADF and ABF STEM images in Figure 2b left and right, respectively. As the (003) facet constitutes only a small fraction of the octahedron surface, our single crystals are predominately enclosed by a spinel surface layer. Similar results were also observed on the LNMO sample (Figure S2), indicating that surface reconstructions are independent of fluorination. The results are also consistent with previous reports on LMRs where a thin surface reconstruction layer was observed on the layer-structured samples made by various methods.^34,41^

Figure 2c,d compares the brightness intensity of the STEM-HAADF images collected on the (102) and (003) facets as well as the corresponding elemental distributions from the STEM-EDX analysis. While the (003) surface shows similar intensity as that in the bulk lattice, the (102) facet shows significantly higher intensity. As the brightness intensity in the STEM-HAADF imaging is roughly proportional to Z^2^ (Z is the atomic number), the results suggest the enrichment of heavier elements on the (102) surface. Further EDX mapping analysis showed uniform distributions of both Mn and Ni in the bulk, whereas Ni is strongly enriched on the top ∼2–3 nm of the (102) facet (Figure 2e). On the other hand, only a slight Ni segregation was observed on the (003) facet (Figure 2f). The results suggest that the surface spinel layer is enriched with Ni (Mn/Ni ratio is <3). Similar results were also obtained on the unfluorinated LNMO sample (Figure S2), indicating that Ni segregation is facet-dependent but independent of the fluorination process.

Aside from the SRL presence on the surface, the spinel-like phase was also found in the bulk of LNMO-F5 crystals, as shown by the STEM imaging and the corresponding fast Fourier transform (FFT) analysis (Figures 2g). In the STEM image, the spinel (yellow square) and layered (blue square) structures once again appear crystallographically coherent without the presence of phase boundaries, suggesting the coherent integration of spinel domains in the native layered framework. The corresponding FFT analysis further confirms the nature of both phases, with the layered indexed as the hexagonal *R*3̅*m* structure and the spinel as the cubic *P*4_3_32 structure. Through EDX analysis, the presence of Ni was detected in both layered and spinel phases. The extent of spinel presence as well as the ratio between Mn and Ni vary depending on the location of the F-LNMO particle. The general formula of the spinel phase is consistent with the HV spinel phase, LiNi_*x*_Mn_2–*x*_O_4_, with *x* > 0.5 (Ni-rich) on the surface.

**Figure 3:**
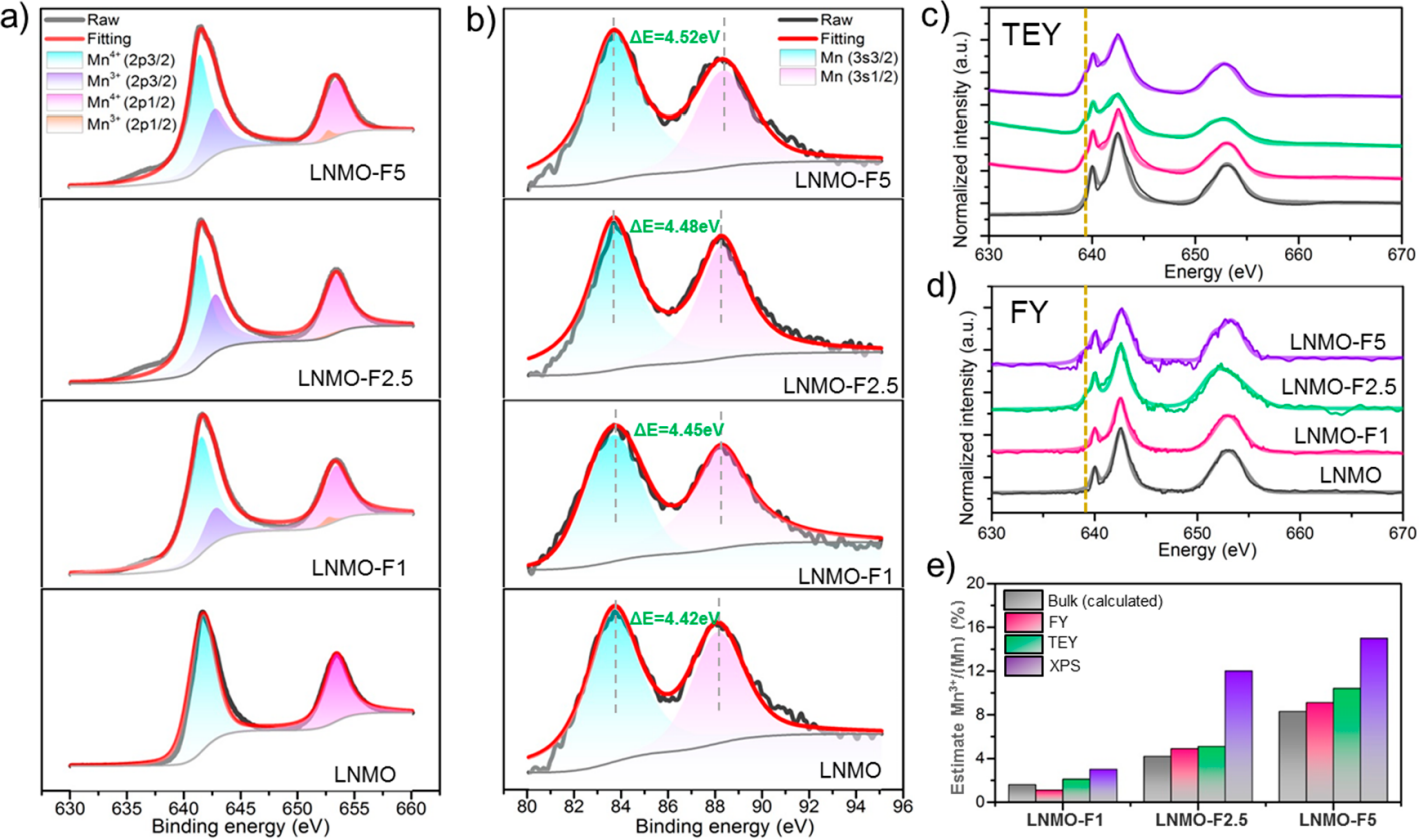
(a) Mn 2p and (b) Mn 3s XPS spectra of the single-crystal samples. Mn *L*-edge soft XAS profiles of the samples collected in (c) TEY and (d) FY mode. (e) Mn^3+^ contents estimated from the various characterization techniques.

We further employed XPS, soft XAS spectroscopy, and EELS to analyze the elemental and chemical distributions in our single-crystal samples. The study aims to probe these distributions covering the entire range of surface to bulk, with the XPS probing roughly 2 nm on the top surface, sXAS in the total electron yield (TEY) mode and fluorescence yield (FY) mode probing ∼5 nm surface and 50 nm subsurface regions, respectively,^42,43^ and the EELS technique combined with FIB lithography probing the bulk region. Figure 3a,b shows the fitted Mn 2p and Mn 3s XPS spectra, respectively. For LNMO, surface Mn cations are at 4+. The presence of lower-valence Mn^3+^ cation was detected on all F-LNMO crystals, with its content increasing with the increasing fluorination level. The Ni oxidation state, on the other hand, remains unchanged for all of the LNMO and F-LNMO samples (Figure S3a). On the F 1s XPS spectra, a broad peak centered around 684 eV was visible on LNMO-F2.5 and LNMO-F5 (Figure S3b), suggesting the presence of F on the sample. This value is much lower compared to the binding energy of LiF (∼685.4 eV),^19^ further confirming the absence of LiF impurity in our SC samples. Mn *L*-edge soft XAS spectra in both the TEY and FY detection modes are shown in Figure 3c,d, respectively. Compared to that of the LNMO sample, the *L*_3_ absorption edges of the F-LNMO samples show an additional shoulder peak at ∼639.5 eV (yellow dashed lines) in both TEY and FY. This is consistent with the presence of Mn^3+^ along with Mn^4+^ in the fluorinated samples, in comparison to the reference spectra collected on various manganese oxide standards (Figure S4). Thus, the formation of Mn^3+^ cations from the very top surface to the subsurface region in the F-LNMO is confirmed by combining XPS and sXAS analyses. We further evaluated the Mn^3+^ distribution by estimating its content from principle component analysis (PCA) of the collected spectra (Figure 3e). Again, the overall Mn^3+^ content increases with fluorination level, with the highest content detected on LNMO-F5. This is consistent with the trend in calculated bulk Mn^3+^ based on charge compensation upon replacing O^2–^ with F^–^ in the lattice. Furthermore, in a given F-LNMO sample, there is clearly a concentration gradient of Mn^3+^ as its content decreases from the top surface to the subsurface region of ∼50 nm.

**Figure 4:**
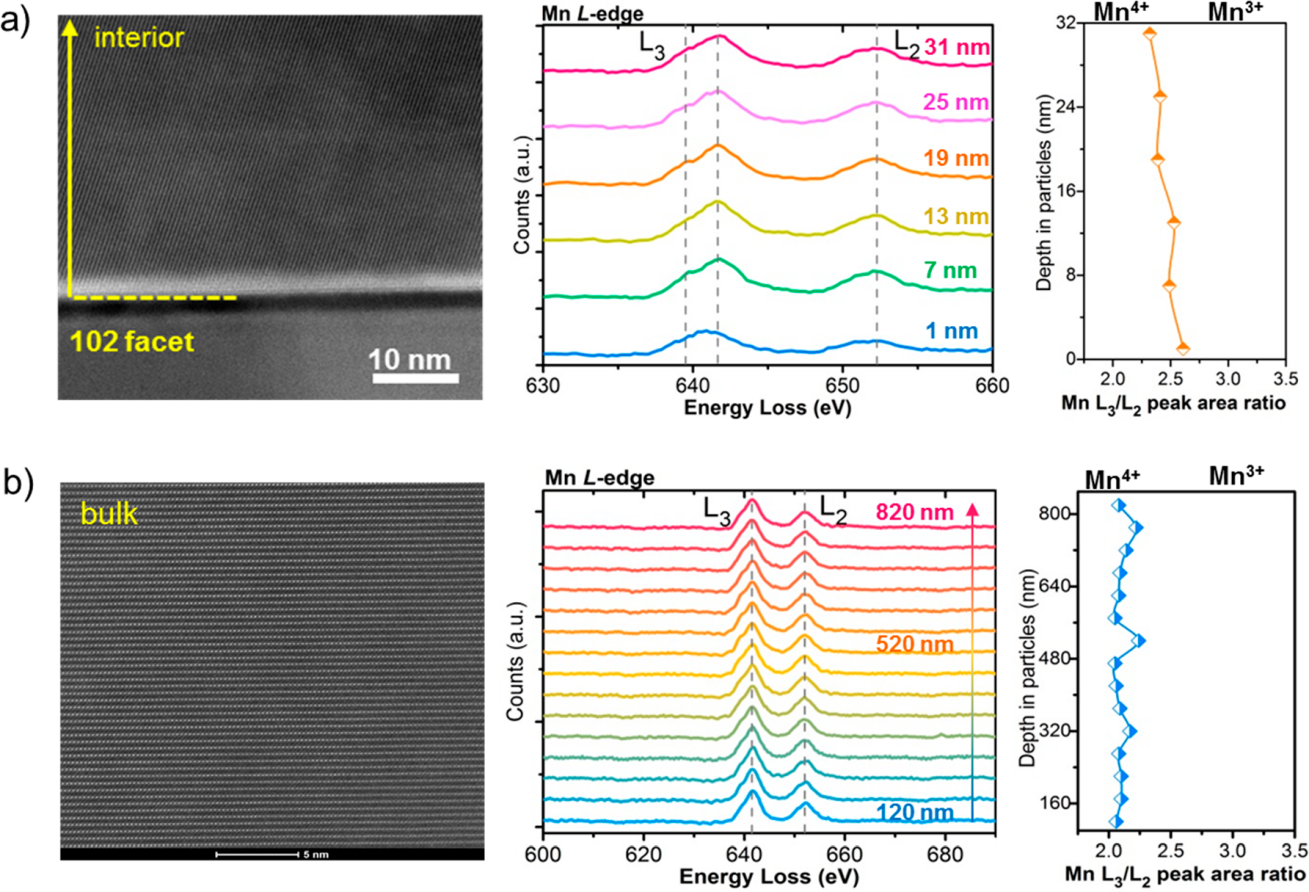
STEM-HAADF and Mn *L*_2_/*L*_3_-edge STEM-EELS spectra collected on an LNMO-F5 crystal probed: (a) in the (102) surface region of 1 to 31 nm at a step size of 6 nm and (b) in the bulk region from 120 to 820 nm at a step size of 50 nm.

The surface to bulk distribution of the Mn oxidation state in the LNMO-F5 crystals was further examined by the STEM-EELS integrated spectroscopy. Figure 4 and Figure S5 show Mn *L*_3_/*L*_2_-edge and O *K*-edge EELS spectra, respectively, both collected in the (102) surface region of 1–31 nm and the bulk region of 120–820 nm. The peak area ratio between the *L*_3_-edge and *L*_2_-edge on the Mn spectra is typically used to determine the valence of Mn cations.^41^ It is clear that the Mn-*L*_3_/*L*_2_ peak area ratios near the (102) surface region (Figure 4a) are broadly higher than that in the bulk region (Figure 4b), consistent with an overall higher Mn^3+^ cation content near the surface. The gradient distribution in the Mn^3+^ content is also confirmed, with the highest content detected near the top surface and lowest content in the bulk region. The results further reveal the particle-level gradient distribution of Mn^3+^ concentration from the surface to bulk of the F-LNMO crystals. Our attempt to spatially resolve F distribution, however, was unsuccessful. In the STEM-EELS analysis, the detected F signals fall into the noise level due to its low concentration. In the STEM-EDX analysis, on the other hand, the F-*K* edge signal overlaps with the Mn-*L* edge signal in the F-LNMO, resulting in difficulties in deconvoluting the signals (Figure S6).

In all, detailed characterization reveals that in LNMO single crystals, the surface is enclosed with a thin layer of spinel LiNi_*x*_Mn_2-x_O_4_ (*x* > 0.5), whereas the bulk remains the layered structure. Upon fluorination, there is a concentration gradient of Mn^3+^ whose content decreases from the top surface to the subsurface region of the F-LNMO particle. This can be attributed to the formation of a Ni-enriched Li_*z*_Ni_*x*_Mn_2–*x*_O_4–*y*_F_*y*_ (*x* > 0.5) phase where Mn^3+^ is generated upon charge compensation. As charge compensation can also be achieved by changes in the Li stoichiometry in this case, it is possible that the Li content (*z*) deviates from 1. Particularly, locally lithium-rich cation disordered environments are known to be enablers for F incorporation into cathode materials such as cation-disordered rocksalts.^44^ Their presence may play a key role in LNMO fluorination, as well. Lower Mn^3+^ content was found in the subsurface, which may result from the formation of spinel LiNi_*x*_Mn_2–*x*_O_4_ (*x* < 0.5) domains or some F incorporation into the spinel and/or the layered structures. On the other hand, bulk Mn remains at 4+, suggesting a “spinel-layered” structure where domains of the LiNi_0.5_Mn_1.5_O_4_ spinel phase are integrated into the native layered framework.

## Electrochemical Performance of F-LNMO Single-Crystal Cathodes

**Figure 5:**
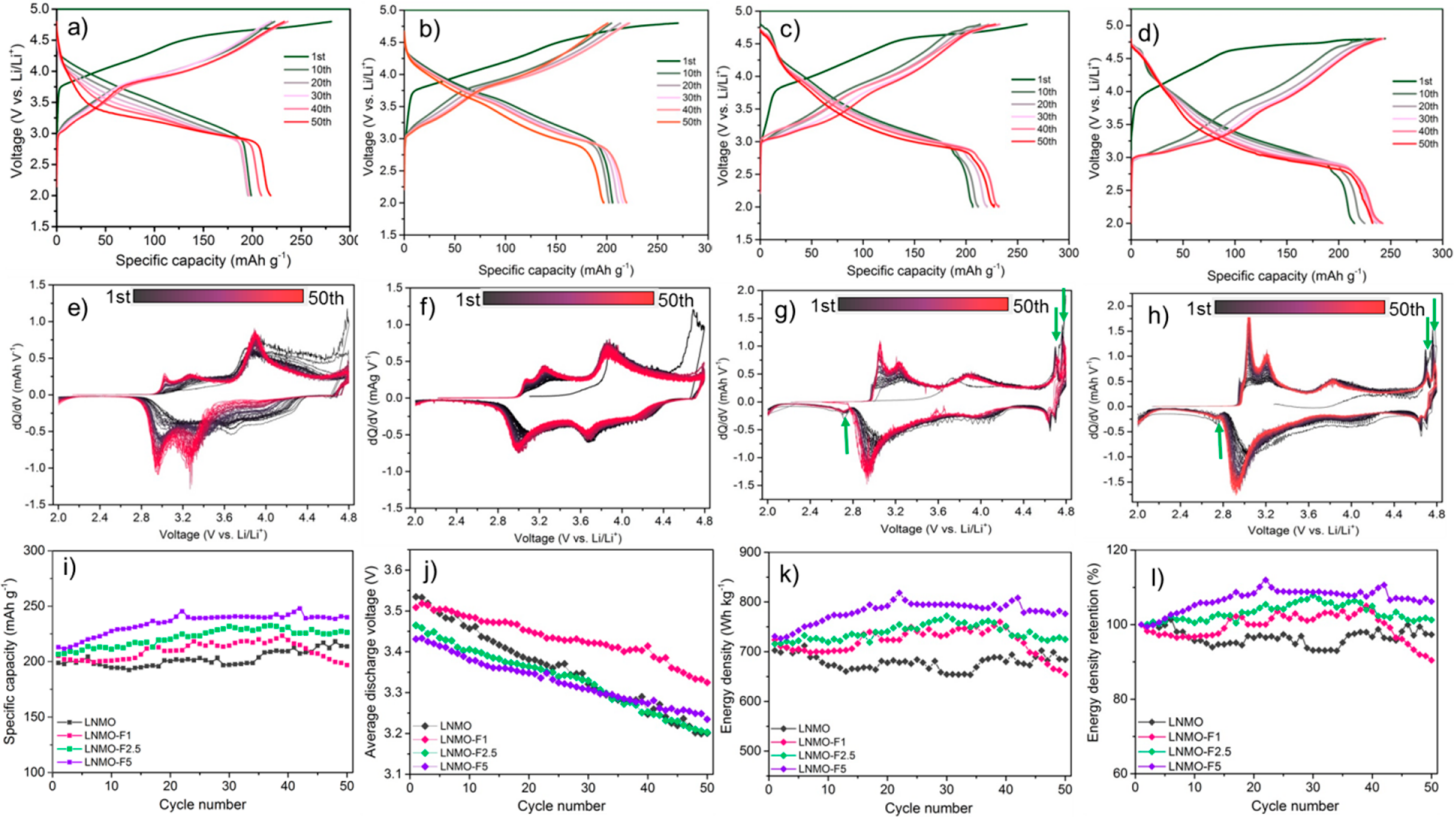
Voltage profiles of (a) LNMO, (b) LNMO-F1, (c) LNMO-F2.5, and (d) LNMO-F5 cathodes at 0.1C in the voltage range of 2–4.8 V. The corresponding d*Q*/d*V* profiles of (e) LNMO, (f) LNMO-F1, (g) LNMO-F2.5, and (h) LNMO-F5 cathodes from the 1st cycle to 50th cycle. Comparison of (i) specific capacities, (j) average discharge voltage, (k) energy density, and (l) energy density retention of LNMO and fluorinated LNMO cathodes.

. Electrochemical performances of the as-synthesized LNMO and F-LNMO cathodes were evaluated in standard half-cell CR 2032-coin cell configuration with a Gen 2 electrolyte (1 M LiPF_6_ in EC/EMC 3:7). Figure 5a–d shows the charge/discharge voltage profiles of the cells when cycled at 0.1C in the voltage window of 2–4.8 V. All samples showed the typical first cycle charge curve with a slopy profile below 4.5 V (vs. Li^+^/Li) and a long plateau above 4.5 V. On discharge, the LNMO and LNMO-F1 samples showed the typical S-shaped profile (Figure 5a,b) while that of LNMO-F2.5 and LNMO-F5 showed additional voltage plateaus centered at ∼2.7, 4.7, and 4.75 V (Figure 5c,d). The differences are clearly shown in the corresponding d*Q* and d*V* profiles (Figure 5e–h). Upon further cycling, the LNMO cathode experienced significant changes in the voltage profile, accompanied by a gradual decrease in average discharge voltage and the formation of a new reduction peak at ∼2.9 V. This is consistent with the cycling-induced layered-to-spinel (LiMn_2_O_4_-type not the HV-spinel) transition previously reported on LMR cathodes. On the other hand, no significant changes were observed on that of F-LNMO cathodes, suggesting that the detrimental phase transition process is suppressed by fluorination.

For LNMO-F2.5 and LNMO-F5, the additional redox processes occurring at ∼2.7, 4.7, and 4.75 V in the first cycle are active in the following cycles, and they continue to contribute to charge storage capacity. These redox couples are known as the signature of high-voltage spinel cathodes (LiNi_*x*_Mn_2–*x*_O_4_). During charge and discharge, the two plateaus separated by approximately 50 mV at 4.7 V are consistent with the two-step extraction/insertion process associated with the 8*a* tetrahedral sites of the cubic spinel structure utilizing the Ni^2+^/Ni^4+^ redox couple. The reaction plateau at ∼2.7 V is associated with the insertion of Li^+^ into the 16*c* octahedral sites of the spinel phase, where lithium ions are displaced from tetrahedral to octahedral sites with the concomitant reduction of Mn from 4+ to 3+.^13,39^ Discharged F-LNMO cathodes, therefore, can be expected to have increased Mn^3+^ content compared to the pristine electrode. The results show that repeated involvement of the HV-spinel component in the as-synthesized F-LNMO provides a stabilizing effect on LMR cycling.

Figure 5i compares the discharge capacities of the LNMO and the F-LNMO cathodes. The initial discharge capacities were ∼201, 206, 210, and 220 mAh g^–1^ for LNMO, LNMO-F1, LNMO-F2.5, and LNMO-F5 cathodes, respectively. We attribute the increased capacity in F-LNMO to the involvement of the LiNi_*x*_Mn_2–*x*_O_4_ HV spinel phase. The presence of Mn^3+^ likely leads to an increase in the electronic conductivity, and the increased amount of the spinel phase enables more Li^+^ accommodation through overlithiation of the spinel phase upon discharge (the theoretical capacity of the overlithiated Li_2_Ni_0.5_Mn_1.5_O_4_ is 282 mAh g^–1^). Furthermore, the spinel phase has faster 3D Li-ions diffusion pathways than the 2D diffusion pathways in the layered structure. When cycled at a given current density, this leads to improved active material utilization and higher cathode capacity. After 50 cycles, the specific discharge capacities changed to ∼210, 198, 226, and 240 mAh g^–1^, with a capacity retention of ∼106%, 97%, 109%, and 114%, respectively (Figure S7a). The increase in capacity is likely due to enhanced ionic and electronic conduction as well as electrolyte wetting upon cycling of the composite cathode, a “break-in” process that has been reported previously.^45,46^ In addition, the initial Coulombic efficiency was also improved in F-LNMO (Figure S7b), reaching 78%, 79%, and 84% for LNMO-F1, LNMO-F2.5, and LNMO-F5, respectively, as compared to 71% for LNMO. Figure 5j and Figure S7c compare the average discharge voltage and the voltage retention. Although the data has not been *iR*-corrected, we believe the comparison within the series is meaningful, as the only variable among different composite cathodes is the fluorination of the active material. All cathodes showed a gradual decrease in average discharge voltage during cycling; however, the extent of decay is significantly reduced in all F-LNMO cathodes. While LNMO-F5 showed the best cycling stability, the LNMO-F1 cathode appears to be most stable against voltage decay, suggesting that voltage decay is only one of the contributors to the overall cycling stability. We also observed improved energy density and energy density retention in the F-LNMO cathodes. After 50 cycles, the values are 725 and 775 Wh kg^–1^ for LNMO-F2.5 and LNMO-F5, respectively, as compared to 695 Wh kg^–1^ for LNMO (Figure 5k). The corresponding energy density retention are 96%, 91%, 102%, and 106% for LNMO, LNMO-F1, LNMO-F2.5, and LNMO-F5, respectively (Figure 5l).

We note that the capacity of our LNMO cathodes is relatively low compared to some of those reported in the literature. This is due to the large micrometer size of the single-crystal particles as opposed to the polycrystalline samples composed of primary particles in tens of nm size. The increased diffusion length in the active particles leads to a lower material utilization and consequently a lower capacity. Nonetheless, our results clearly demonstrate that excellent performance can be achieved even on large micrometer-sized LNMO particles. Fluorination has a positive effect on the electrochemical performance of LNMO single-crystal cathodes, with improvement achieved in nearly all performance metrics, including discharge capacity and capacity retention, Coulombic efficiency, average discharge voltage and voltage retention, energy density, and energy density retention. We wish to point out that a large fraction of charge storage capacity in LNMO-F2.5 and LNMO-F5 cathodes involves the Mn^3+/^Mn^4+^ redox and the Ni^2+^/Ni^4+^ redox above 4.5 V, increasing the energy output of the cathodes. This is in contrast to what is observed in traditional LMR cathodes where the anionic redox occurring above 4.5 V leads to an O loss and the involvement of Mn^3+^/Mn^4+^ redox is associated with the undesirable layered-to-spinel transformation, both of which cause capacity and voltage decay.

Furthermore, we note that the effect of fluorination level on LMR performance is likely exacerbated on these micrometer-sized single crystals. Previous studies have shown that in nanosized LMR, significant performance improvement can be achieved through surface focused fluorine treatments such as surface fluorine coating or electrolyte fluorination.^25,47,48^ It is possible that high fluorination levels do not have the same impact on small LMR particles with much larger surface areas. However, large microsized particles have many advantages over nanoparticles, and approaches to enable their use as cathode materials are especially attractive for developing next-generation LIB systems.

## Understanding the Fluorination Effect on LNMO Single-Crystal Cathodes

. To understand the fluorination effect on LMR, we carried out a range of post-mortem analyses on the cycled electrodes. Discharged LNMO and LNMO-F5 cathodes recovered after various cycle numbers were analyzed by synchrotron XRD (Figure S8). For the pristine electrodes, only the layered phase was detected on the LNMO cathode, while both spinel and layered phases were found on the LNMO-F5 cathode. The recovered cathodes showed that the spinel phase remains nearly unchanged in its peak position and intensity during the initial cycling of the LNMO-F5 cathode. On the other hand, the discharged LNMO cathodes showed a newly formed spinel phase which gradually increased its content with cycling. Compared to the preformed HV spinel in LNMO-F5, the spinel peaks that appeared during LNMO cycling are much broader and they have lower intensity, corresponding to *in situ* generated small spinel domains.

**Figure 6:**
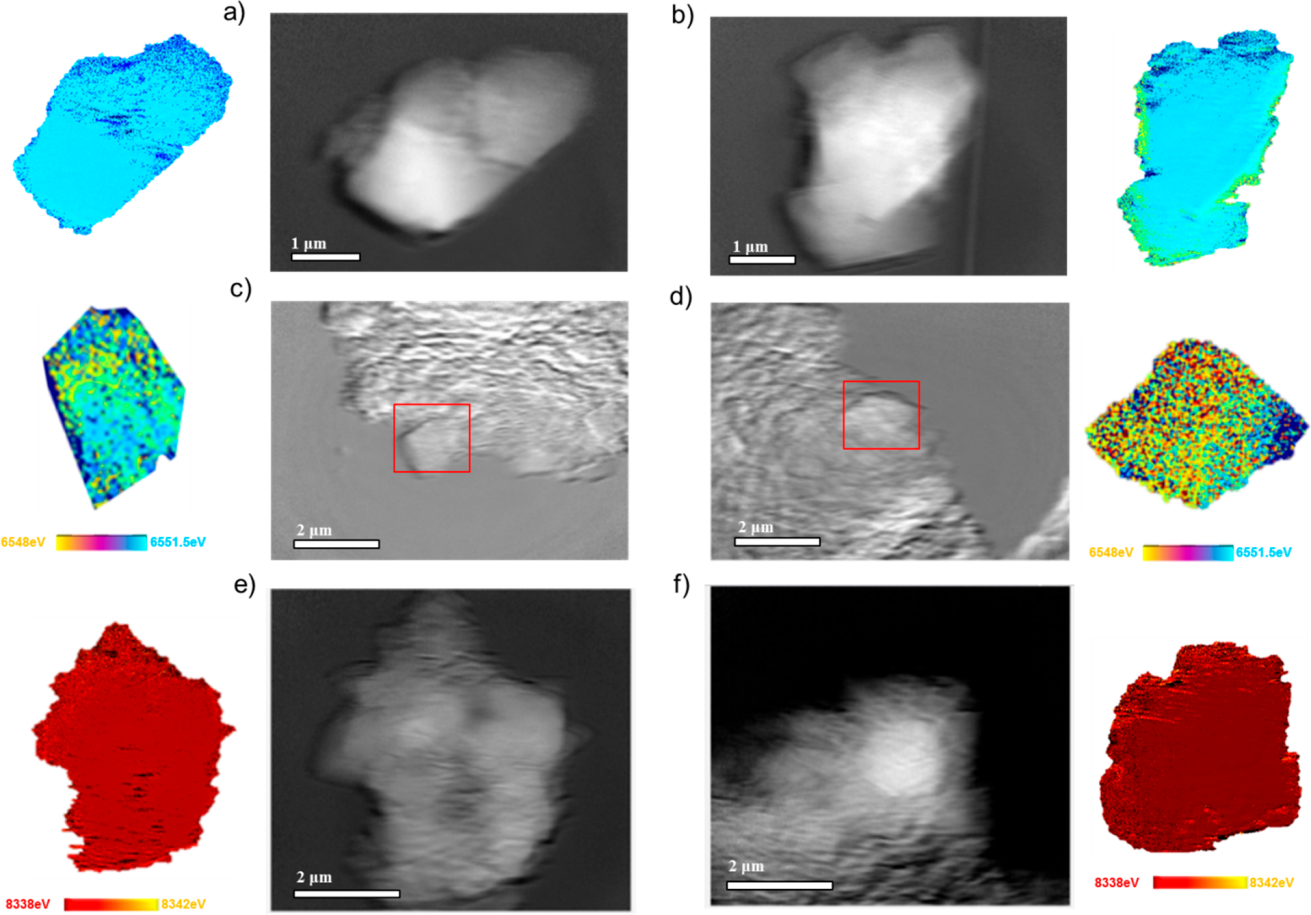
TXM images and the corresponding 2D nanoscale Mn *K*-edge imaging of (a) pristine LNMO, (b) pristine LNMO-F5, (c) discharged LNMO recovered after 10 cycles, and (d) discharged LNMO-F5 recovered after 10 cycles. TXM images and the corresponding 2D nanoscale Ni *K*-edge imaging of (e) discharged LNMO recovered after 10 cycles, and (f) discharged LNMO-F5 recovered after 10 cycles.

We further compared the particle-level chemical distribution of Ni and Mn before cycling and after 10 cycles using hard X-ray full-field transition microscopy imaging combined with the X-ray absorption near-edge structure (FF-TXM-XANES) technique. The brightness and the energy tunability of synchrotron-based hard X-ray enable nanoscale spatial resolution at ∼30 nm along with high chemical and elemental sensitivities in a large field-of-view (FOV, 30 μm × 30 μm). Figure 6a,b shows the TXM images and the corresponding 2D nanoscale Mn and Ni *K*-edge imaging of the pristine LNMO and LNMO-F5 cathodes, respectively. The 2D chemical maps were generated by linear combination fitting of the standard XANES spectra (Figure S9), which are color-coded according to the color legend shown in the figure. In the raw tomography images, LNMO and LNMO-F5 electrodes contained large areas of conductive carbon additive or binder, which were difficult to separate from the active material. Nonetheless, lateral chemical heterogeneity in Mn oxidation states was clearly visible in the 2D Mn *K*-edge energy distribution map of LNMO-F5, with the presence of Mn^3+^ on the surface and Mn^4+^ only in the bulk (Figure 6b). On the other hand, the Mn oxidation state is at 4+ throughout the LNMO particles (Figure 6a), which is consistent with the results of Mn *L*-edge spectra in s-XAS and EELS data. After cycling, a small percentage of low-valence Mn^3+^ cations are present on LNMO (Figure 6c), corresponding to the layered-to-spinel transformation during cycling. For LNMO-F5, there is a large increase in Mn^3+^ content after 10 cycles, confirming the overlithiation of the spinel phase upon discharge which is accompanied by the formation of Mn^3+^ in the structure (Figure 6d).^27,49^ In addition, the Ni oxidation state for LNMO and LNMO-F5 particles remains at 2+ after 10 cycles (Figure 6e,f), demonstrating that the Ni redox process is highly reversible in both cases.

**Figure 7:**
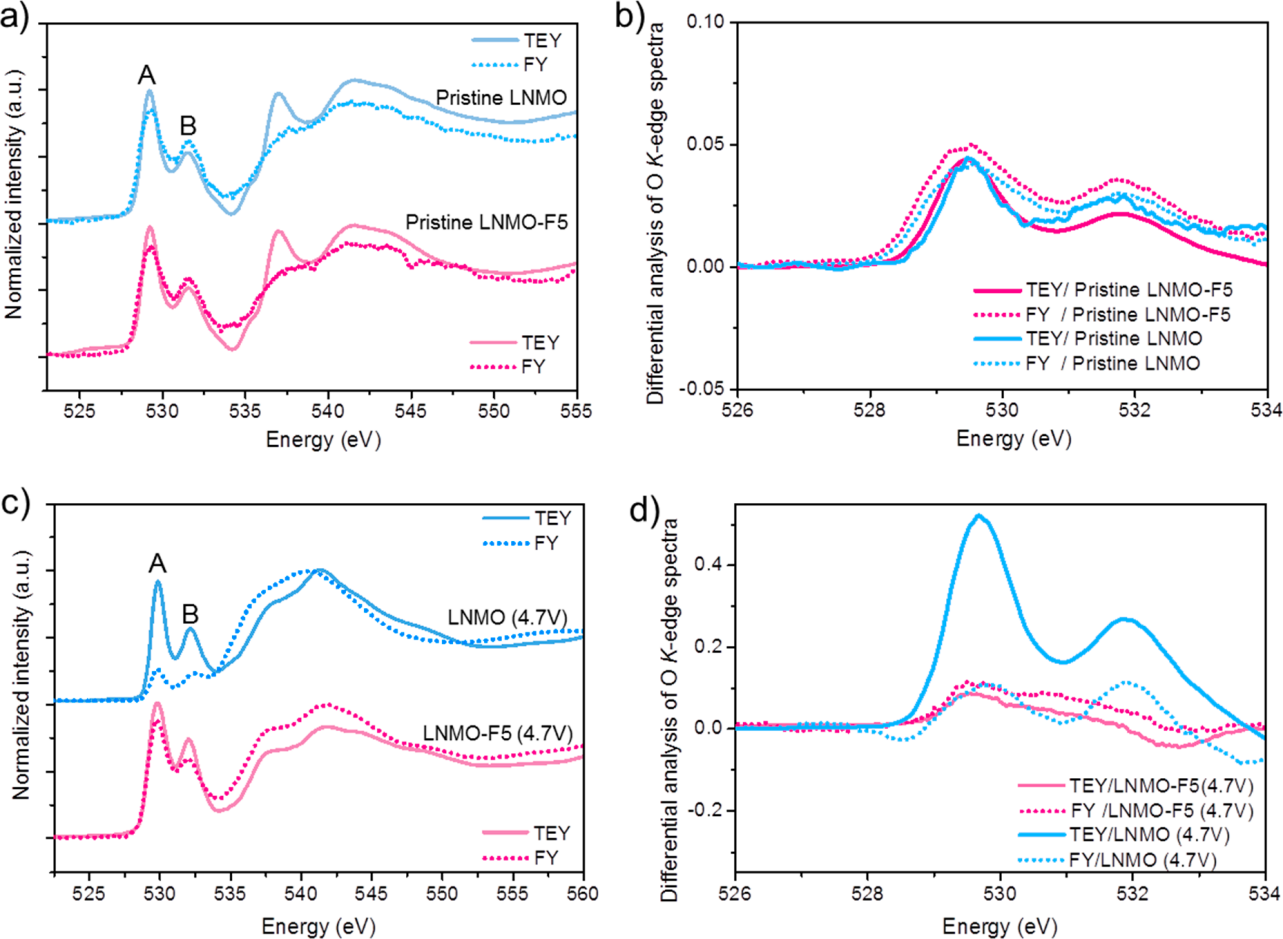
Normalized O *K*-edge XAS spectra and the corresponding differential spectral analysis of (a, b) pristine LNMO and LNMO-F5 and (c, d) recovered LNMO and LNMO-F5 cathodes after charging to 4.7 V. (b) and (d) are expanded views of the pre-edge regions in (a) and (c), respectively.

O *K*-edge soft X-ray spectroscopy was also used to compare oxygen activities in the LNMO and LNMO-F2.5 electrodes. The TEY and FY spectra of both pristine electrodes are similar (Figure 7a,b). The spectra display two regions: (1) a pre-edge between 525–535 eV corresponding to the O_1s_ to TM_3d_-O_2p_ transition, where the pre-edge peak A at 529.6 eV comes from the excitation of O 1s electrons to the hybridized O 2p-t2g orbitals and the pre-edge peak B at 531.9 eV is attributed to the hybridized O 2p-eg orbitals,^2,50,51^ and (2) a broad peak above 535 eV arising from the O_1s_ to TM_4s4p_-O_2p_ transitions. The pre-edge region is of greater importance as it relates to the hybridization between TM and O. The changes in the pre-edge intensity are therefore associated with the changes in TM valence and oxygen activities. Figure 7c,d show the TEY and FY spectra obtained on charged cathodes after the initial cycles, respectively. The TM_3d_-O_2p_ pre-edge intensity of LNMO in the TEY mode is higher than that in FY mode (Figure 7c), indicating enhanced hybridization of TM_3d_-O_2p_ on the surface than that in the subsurface.^6,50^ Considering minimum changes on Mn and Ni oxidation states (Figure 3 and Figure S3), the differences suggest enhanced O activities on the LNMO surface compared to the subsurface region. On the other hand, there are no significant changes in the pre-edge region of the TEY and FY spectra collected on the charged LNMO-F5 cathode, suggesting a similar level of TM_3d_-O_2p_ hybridization. This is further shown by comparing the TEY and FY O *K*-edge XAS spectra peak features in both LNMO and LNMO-F5 (Figure 7d).

The oxygen redox activities in LMR cathodes are known to involve both the reversible redox process and irreversible oxygen release. While the reversible oxygen redox typically occurs in the bulk lattice, oxygen release only occurs on the surface.^7,9,10^ TM_3d_-O_2p_ pre-edge intensity in the FY spectra of LNMO and LNMO-F5 cathodes is at a similar level, suggesting similar O activities in the subsurface region. Compared to that in LNMO, the lower TEY TM_3d_-O_2p_ pre-edge intensity in LNMO-F5 is likely associated with reduced O_2_ release from the surface. This was confirmed by operando differential electrochemical mass spectrometry (DEMS) analysis. Figure S10 shows the evolution of the O_2_ and CO_2_ gases during the first two cycles of the LNMO and LNMO-F5 cathodes. A small amount of O_2_ gas (3.9 μmol g^–1^) was detected during the first cycle of LNMO, whereas negligible O_2_ evolution was found on the LNMO-F5 cathode. Notably, a large amount of CO_2_ (118.4 μmol g^–1^) evolution was detected during the first cycle as well as the following cycle of the former, whereas CO_2_ evolution was not detected in the latter. As the main source of CO_2_ generation is from the side reactions between the cathode and the carbonate-based electrolyte, our results indicate that fluorination not only minimizes the irreversible oxygen loss from the surface but also reduces the detrimental side reactions at high voltages. This is consistent with the results from a recent study where gradient-fluorination was found to induce a uniform deposition of a thin but robust LiF-enriched cathode-electrolyte interphase (CEI) layer, which provides protection for the cathode surface.^47^

In summary, by variation of the fluoroacidity of the fluorine source, phase-pure fluorinated-LNMO single crystals were successfully synthesized using a modified molten-salt synthesis technique. STEM-EELS analysis reveals that the dominating facets of the single-crystal octahedral particles are (012)-family facets. We show that fluorination improves the specific capacity and capacity retention, Coulombic efficiency, average voltage, and voltage retention as well as energy density and energy retention of the LMR cathodes. We further use the detected surface-to-bulk Mn^3+^ concentration variation to decipher the reasons behind the improvement. Mn^3+^ cations were generated as a result of charge balance from the fluorine incorporation into the spinel lattice and the formation of Ni-rich Li_*z*_Ni_*x*_Mn_2–*x*_O_4–*y*_F_*y*_ (*x* > 0.5) on the surface. The bulk is composed of a “spinel-layered” coherent structure, where domains of a LiNi_0.5_Mn_1.5_O_4_ high-voltage spinel phase are integrated into the native layered framework. We believe the performance enhancement in F-LNMO cathodes is related to the synergic effect of fluorine incorporation and the presence of the high-voltage LiNi_*x*_Mn_*y*_O_4_ spinel phase in the layered structure, both of which improve the structural stability of the LMR cathode.

## Data Availability

The data that support the findings of this study are available in the main text or the Supporting Information of this article.

## Supporting Information Available

Experimental method, XRD patterns of samples, STEM-HAADF and STEM-ABF images, STEM-EDX elemental mapping, XPS, soft XAS, XANES, and STEM-EELS spectra, electrochemical cycling data, gas evolution from *operando* DEMS analysis, properties of F-containing salts used in the study, sample compositions from ICP measurements, supplementary references (PDF)

## References

[ref1] Liu T.; Liu J.; Li L.; Yu L.; Diao J.; Zhou T.; Li S.; Dai A.; Zhao W.; Xu S.; Ren Y.; Wang L.; Wu T.; Qi R.; Xiao Y.; Zheng J.; Cha W.; Harder R.; Robinson I.; Wen J.; Lu J.; Pan F.; Amine K. Origin of Structural Degradation in Li-Rich Layered Oxide Cathode. Nature 2022, 606, 305–312. 10.1038/s41586-022-04689-y. 35676429

[ref2] Li Q.; Ning D.; Wong D.; An K.; Tang Y.; Zhou D.; Schuck G.; Chen Z.; Zhang N.; Liu X. Improving the Oxygen Redox Reversibility of Li-Rich Battery Cathode Materials via Coulombic Repulsive Interactions Strategy. Nat. Commun. 2022, 13, 1123 10.1038/s41467-022-28793-9. 35236854 PMC8891320

[ref3] Eum D.; Kim B.; Kim S. J.; Park H.; Wu J.; Cho S. P.; Yoon G.; Lee M. H.; Jung S. K.; Yang W.; Seong W. M.; Ku K.; Tamwattana O.; Park S. K.; Hwang I.; Kang K. Voltage Decay and Redox Asymmetry Mitigation by Reversible Cation Migration in Lithium-Rich Layered Oxide Electrodes. Nat. Mater. 2020, 19, 419–427. 10.1038/s41563-019-0572-4. 31959949

[ref4] Yan P.; Zheng J.; Tang Z. K.; Devaraj A.; Chen G.; Amine K.; Zhang J. G.; Liu L. M.; Wang C. Injection of Oxygen Vacancies in the Bulk Lattice of Layered Cathodes. Nat. Nanotechnol. 2019, 14, 602–608. 10.1038/s41565-019-0428-8. 31011218

[ref5] Zuo Y.; Shang H.; Hao J.; Song J.; Ning F.; Zhang K.; He L.; Xia D. Regulating the Potential of Anion Redox to Reduce the Voltage Hysteresis of Li-Rich Cathode Materials. J. Am. Chem. Soc. 2023, 145, 5174–5182. 10.1021/jacs.2c11640. 36757130

[ref6] Gent W. E.; Lim K.; Liang Y.; Li Q.; Barnes T.; Ahn S. J.; Stone K. H.; McIntire M.; Hong J.; Song J. H.; Li Y.; Mehta A.; Ermon S.; Tyliszczak T.; Kilcoyne D.; Vine D.; Park J. H.; Doo S. K.; Toney M. F.; Yang W.; Prendergast D.; Chueh W. C. Coupling between Oxygen Redox and Cation Migration Explains Unusual Electrochemistry in Lithium-Rich Layered Oxides. Nat. Commun. 2017, 8, 2091 10.1038/s41467-017-02041-x. 29233965 PMC5727078

[ref7] Hu E.; Yu X.; Lin R.; Bi X.; Lu J.; Bak S.; Nam K. W.; Xin H. L.; Jaye C.; Fischer D. A.; Amine K.; Yang X. Q. Evolution of Redox Couples in Li- and Mn-Rich Cathode Materials and Mitigation of Voltage Fade by Reducing Oxygen Release. Nat. Energy 2018, 3, 690–698. 10.1038/s41560-018-0207-z.

[ref8] Csernica P. M.; Kalirai S. S.; Gent W. E.; Lim K.; Yu Y. S.; Liu Y.; Ahn S. J.; Kaeli E.; Xu X.; Stone K. H.; Marshall A. F.; Sinclair R.; Shapiro D. A.; Toney M. F.; Chueh W. C. Persistent and Partially Mobile Oxygen Vacancies in Li-Rich Layered Oxides. Nat. Energy 2021, 6, 642–652. 10.1038/s41560-021-00832-7.

[ref9] Chen Q.; Pei Y.; Chen H.; Song Y.; Zhen L.; Xu C. Y.; Xiao P.; Henkelman G. Highly Reversible Oxygen Redox in Layered Compounds Enabled by Surface Polyanions. Nat. Commun. 2020, 11, 3411 10.1038/s41467-020-17126-3. 32641725 PMC7343805

[ref10] Zhang J.; Zhang Q.; Wong D.; Zhang N.; Ren G.; Gu L.; Schulz C.; He L.; Yu Y.; Liu X. Addressing Voltage Decay in Li-Rich Cathodes by Broadening the Gap between Metallic and Anionic Bands. Nat. Commun. 2021, 12, 3071 10.1038/s41467-021-23365-9. 34031408 PMC8144552

[ref11] Zuo W.; Luo M.; Liu X.; Wu J.; Liu H.; Li J.; Winter M.; Fu R.; Yang W.; Yang Y. Li-Rich Cathodes for Rechargeable Li-Based Batteries: Reaction Mechanisms and Advanced Characterization Techniques. Energy Environ. Sci. 2020, 13, 4450–4497. 10.1039/D0EE01694B.

[ref12] Zhang B.; Zhang Y.; Wang X.; Liu H.; Yan Y.; Zhou S.; Tang Y.; Zeng G.; Wu X.; Liao H. G.; Qiu Y.; Huang H.; Zheng L.; Xu J.; Yin W.; Huang Z.; Xiao Y.; Xie Q.; Peng D. L.; Li C.; Qiao Y.; Sun S. G. Role of Substitution Elements in Enhancing the Structural Stability of Li-Rich Layered Cathodes. J. Am. Chem. Soc. 2023, 145, 8700–8713. 10.1021/jacs.3c01999. 37029335

[ref13] Zhao S.; Yan K.; Zhang J.; Sun B.; Wang G. Reaction Mechanisms of Layered Lithium-Rich Cathode Materials for High-Energy Lithium-Ion Batteries. Angew. Chem., Int. Ed. 2021, 60, 2208–2220. 10.1002/anie.202000262. 32067325

[ref14] Kang S. H.; Amine K. Layered Li(Li_0.2_Ni_0.15+0.5z_Co_0.10_Mn_0.55–0.5z_)O_2–z_F_z_ Cathode Materials for Li-Ion Secondary Batteries. J. Power Sources 2005, 146, 654–657. 10.1016/j.jpowsour.2005.03.152.

[ref15] Vanaphuti P.; Bai J.; Ma L.; Ehrlich S.; Kisslinger K.; Wang F.; Wang Y. Unraveling Na and F Coupling Effects in Stabilizing Li, Mn-Rich Layered Oxide Cathodes via Local Ordering Modification. Energy Storage Mater. 2020, 31, 459–469. 10.1016/j.ensm.2020.08.003.

[ref16] Wang Y.; Gu H. T.; Song J. H.; Feng Z. H.; Zhou X. B.; Zhou Y. N.; Wang K.; Xie J. Y. Suppressing Mn Reduction of Li-Rich Mn-Based Cathodes by F Doping for Advanced Lithium-Ion Batteries. J. Phys. Chem. C 2018, 122, 27836–27842. 10.1021/acs.jpcc.8b08669.

[ref17] Pang W. K.; Lin H. F.; Peterson V. K.; Lu C. Z.; Liu C. E.; Liao S. C.; Chen J. M. Effects of Fluorine and Chromium Doping on the Performance of Lithium-Rich Li_1+x_MO_2_ (M = Ni, Mn, Co) Positive Electrodes. Chem. Mater. 2017, 29, 10299–10311. 10.1021/acs.chemmater.7b02930.

[ref18] Pei Y.; Li S.; Chen Q.; Liang R.; Li M.; Gao R.; Ren D.; Deng Y. P.; Jin H.; Wang S.; Su D.; Hu Y.; Chen Z. Cationic–Anionic Redox Couple Gradient to Immunize Against Irreversible Processes of Li-Rich Layered Oxides. J. Mater. Chem. A 2021, 9, 2325–2333. 10.1039/D0TA09609A.

[ref19] Luo D.; Ding X.; Fan J.; Zhang Z.; Liu P.; Yang X.; Guo J.; Sun S.; Lin Z. Accurate Control of Initial Coulombic Efficiency for Lithium-Rich Manganese-Based Layered Oxides by Surface Multicomponent Integration. Angew. Chem., Int. Ed. 2020, 59, 23061–23066. 10.1002/anie.202010531. 32820858

[ref20] Ménétrier M.; Bains J.; Croguennec L.; Flambard A.; Bekaert E.; Jordy C.; Biensan Ph.; Delmas C. NMR Evidence of LiF Coating Rather than Fluorine Substitution in Li(Ni_0.425_Mn_0.425_Co_0.15_)O_2_. J. Solid State Chem. 2008, 181, 3303–3307. 10.1016/j.jssc.2008.09.002.

[ref21] Croguennec L.; Bains J.; Ménétrier M.; Flambard A.; Bekaert E.; Jordy C.; Biensan P.; Delmas C. Synthesis of “Li_1.1_(Ni_0.425_Mn_0.425_Co_0.15_)_0.9_O_1.8_F_0.2_” Materials by Different Routes: Is There Fluorine Substitution for Oxygen?. J. Electrochem. Soc. 2009, 156, A349–A355. 10.1149/1.3080659.

[ref22] Liu H.; Qian D.; Verde M. G.; Zhang M.; Baggetto L.; An K.; Chen Y.; Carroll K. J.; Lau D.; Chi M.; Veith G. M.; Meng Y. S. Understanding the Role of NH_4_F and Al_2_O_3_ Surface Co-Modification on Lithium-Excess Layered Oxide Li_1.2_Ni_0.2_Mn_0.6_O_2_. ACS Appl. Mater. Interfaces 2015, 7, 19189–19200. 10.1021/acsami.5b04932. 26287963

[ref23] Richards W. D.; Dacek S. T.; Kitchaev D. A.; Ceder G. Fluorination of Lithium-Excess Transition Metal Oxide Cathode Materials. Adv. Energy Mater. 2018, 8, 1701533 10.1002/aenm.201701533.

[ref24] Gao C.; Zhou J.; Liu G.; Wang L. Synthesis of F-doped LiFePO_4_/C Cathode Materials for High Performance Lithium-Ion Batteries using Co-Precipitation Method with Hydrofluoric Acid Source. J. Alloys Compd. 2017, 727, 501–513. 10.1016/j.jallcom.2017.08.149.

[ref25] Jiang Y. S.; Sun G.; Yu F. D.; Que L. F.; Deng L.; Meng X. H.; Wang Z. B. Surface Modification by Fluorine Doping to Increase Discharge Capacity of Li_1.2_Ni_0.2_Mn_0.6_O_2_ Cathode Materials. Ionics 2020, 26, 151–161. 10.1007/s11581-019-03202-2.

[ref26] Park S.-H.; Kang S.-H.; Johnson C. S.; Amine K.; Thackeray M. M. Lithium–Manganese–Nickel-Oxide Electrodes with Integrated Layered–Spinel Structures for Lithium Batteries. Electrochem. Commun. 2007, 9, 262–268. 10.1016/j.elecom.2006.09.014.

[ref27] Kim D.; Sandi G.; Croy J. R.; Gallagher K. G.; Kang S. H.; Lee E.; Slater M. D.; Johnson C. S.; Thackeray M. M. Composite ‘Layered-Layered-Spinel’ Cathode Structures for Lithium-Ion Batteries. J. Electrochem. Soc. 2013, 160, A31–A38. 10.1149/2.049301jes.

[ref28] Long B. R.; Croy J. R.; Park J. S.; Wen J.; Miller D. J.; Thackeray M. M. Advances in Stabilizing ‘Layered-Layered’ xLi_2_MnO_3_•(1-x)LiMO_2_ (M = Mn, Ni, Co) Electrodes with a Spinel Component. J. Electrochem. Soc. 2014, 161, A2160–A2167. 10.1149/2.0681414jes.

[ref29] Chen S.; Xie Y.; Chen W.; Chen J.; Yang W.; Zou H.; Lin Z. Enhanced Electrochemical Performance of Li-Rich Cathode Materials by Organic Fluorine Doping and Spinel Li_1+x_Ni_y_Mn_2–y_O_4_ Coating. ACS Sustainable Chem. Eng. 2020, 8, 121–128. 10.1021/acssuschemeng.9b04665.

[ref30] Wu T.; Zhang X.; Wang Y.; Zhang N.; Li H.; Guan Y.; Xiao D.; Liu S.; Yu H. Gradient “Single-Crystal” Li-Rich Cathode Materials for High-Stable Lithium-Ion Batteries. Adv. Funct. Mater. 2023, 33, 2210154 10.1002/adfm.202210154.

[ref31] Zhu Z.; Yu D.; Yang Y.; Su C.; Huang Y.; Dong Y.; Waluyo I.; Wang B.; Hunt A.; Yao X.; Lee J.; Xue W.; Li J. Gradient Li-Rich Oxide Cathode Particles Immunized Against Oxygen Release by a Molten Salt Treatment. Nat. Energy 2019, 4, 1049–1058. 10.1038/s41560-019-0508-x.

[ref32] Zeng W.; Liu F.; Yang J.; Zhang B.; Cao F.; Tian W.; Wang J.; Yu R.; Xia F.; Peng H.; Ma J.; Wang Z.; Mu S.; Wu J. Single-Crystal Li-Rich Layered Cathodes with Suppressed Voltage Decay by Double-Layer Interface Engineering. Energy Storage Mater. 2023, 54, 651–1058. 10.1016/j.ensm.2022.11.016.

[ref33] Zhang T.; Li J. T.; Liu J.; Deng Y. P.; Wu Z. G.; Yin Z. W.; Guo D.; Huang L.; Sun S. G. Suppressing the Voltage-Fading of Layered Lithium-Rich Cathode Materials via an Aqueous Binder for Li-Ion Batteries. Chem. Commun. 2016, 52, 4683–4686. 10.1039/C5CC10534J. 26954264

[ref34] Kuppan S.; Shukla A. K.; Membreno D.; Nordlund D.; Chen G. Revealing Anisotropic Spinel Formation on Pristine Li- and Mn-Rich Layered Oxide Surface and its Impact on Cathode Performance. Adv. Energy Mater. 2017, 7, 1602010 10.1002/aenm.201602010.

[ref35] Bieber A. L.; Massot L.; Gibilaro M.; Cassayre L.; Chamelot P.; Taxil P. Fluoroacidity Evaluation in Molten Salts. Electrochim. Acta 2011, 56, 5022–5027. 10.1016/j.electacta.2011.03.099.

[ref36] Zhao H.; Li W.; Li J.; Xu H.; Zhang C.; Li J.; Han C.; Li Z.; Chu M.; Qiu X. Enhance Performances of Co-Free Li-Rich Cathode by Eutesctic Melting Salt Treatment. Nano Energy 2022, 92, 106760 10.1016/j.nanoen.2021.106760.

[ref37] Luo K.; Roberts M. R.; Hao R.; Guerrini N.; Liberti E.; Allen C. S.; Kirkland A. I.; Bruce P. G. One-Pot Synthesis of Lithium-Rich Cathode Material with Hierarchical Morphology. Nano Lett. 2016, 16, 7503–7508. 10.1021/acs.nanolett.6b03296. 27792340

[ref38] Li X.; Qiao Y.; Guo S.; Xu Z.; Zhu H.; Zhang X.; Yuan Y.; He P.; Ishida M.; Zhou H. Direct Visualization of the Reversible O^2–^/O^–^ Redox Process in Li-Rich Cathode Materials. Adv. Mater. 2018, 30, 1705197 10.1002/adma.201705197. 29457283

[ref39] Cui Z.; Zou F.; Celio H.; Manthiram A. Paving Pathways Toward Long-Life Graphite/LiNi_0.5_Mn_1.5_O_4_ Full Cells: Electrochemical and Interphasial Points of View. Adv. Funct. Mater. 2022, 32, 2203779 10.1002/adfm.202203779.

[ref40] Lee H. J.; Liu X.; Chart Y.; Tang P.; Bae J. G.; Narayanan S.; Lee J. H.; Potter R. J.; Sun Y.; Pasta M. LiNi_0.5_Mn_1.5_O_4_ Cathode Microstructure for All-Solid-State Batteries. Nano Lett. 2022, 22, 7477–7483. 10.1021/acs.nanolett.2c02426. 36069205 PMC9523706

[ref41] Yan P.; Zheng J.; Zheng J.; Wang Z.; Teng G.; Kuppan S.; Xiao J.; Chen G.; Pan F.; Zhang J. G.; Wang C. M. Ni and Co Segregations on Selective Surface Facets and Rational Design of Layered Lithium Transition-Metal Oxide Cathodes. Adv. Energy Mater. 2016, 6, 1502455 10.1002/aenm.201502455.

[ref42] Lin F.; Markus I. M.; Nordlund D.; Weng T. C.; Asta M. D.; Xin H. L.; Doeff M. M. Surface Reconstruction and Chemical Evolution of Stoichiometric Layered Cathode Materials for Lithium-Ion Batteries. Nat. Commun. 2014, 5, 3529 10.1038/ncomms4529. 24670975

[ref43] Tan S.; Shadike Z.; Li J.; Wang X.; Yang Y.; Lin R.; Cresce A.; Hu J.; Hunt A.; Waluyo I.; Ma L.; Monaco F.; Cloetens P.; Xiao J.; Liu Y.; Yang X. Q.; Xu K.; Hu E. Additive Engineering for Robust Interphases to Stabilize High-Ni Layered Structures at Ultra-High Voltage of 4.8 V. Nat. Energy 2022, 7, 484–494. 10.1038/s41560-022-01020-x.

[ref44] Lee J.; Urban A.; Li X.; Su D.; Hautier G.; Ceder G. Unlocking the Potential of Cation-Disordered Oxides for Rechargeable Lithium Batteries. Science 2014, 343, 519 10.1126/science.1246432. 24407480

[ref45] Liu D.; Fan X.; Li Z.; Liu T.; Sun M.; Qian C.; Ling M.; Liu Y.; Liang C. A Cation/Anion Co-Doped Li_1.12_Na_0.08_Ni_0.2_Mn_0.6_O_1.95_F_0.05_ Cathode for Lithium Ion Batteries. Nano Energy 2019, 58, 786–796. 10.1016/j.nanoen.2019.01.080.

[ref46] Takahashi I.; Kiuchi H.; Ohma A.; Fukunaga T.; Matsubara E. Cathode Electrolyte Interphase Formation and Electrolyte Oxidation Mechanism for Ni-Rich Cathode Materials. J. Phys. Chem. C 2020, 124, 9243–9248. 10.1021/acs.jpcc.0c02198.

[ref47] Lu D.; Chen Y.; Sun W.; Xie W.; Yi S.; Luo S.; Zuo L.; Zhao Y.; Yang T.; Xiao P.; Zheng C. Cathode Electrolyte Interface Engineering by Gradient Fluorination for High-Performance Lithium Rich Cathode. Adv. Energy Mater. 2023, 13, 2301765 10.1002/aenm.202301765.

[ref48] Wang B.; Cui J.; Li Z.; Wang H.; Zhang D.; Wang Q.; Sun H.; Hu Z. Surface F-Doping for Stable Structure and High Electrochemical Performance of Li-Rich Mn-Based Cathode Materials. J. Alloys Compd. 2022, 929, 167304 10.1016/j.jallcom.2022.167304.

[ref49] Xu J.; Sun M.; Qiao R.; Renfrew S. E.; Ma L.; Wu T.; Hwang S.; Nordlund D.; Su D.; Amine K.; Lu J.; McCloskey B. D.; Yang W.; Tong W. Elucidating Anionic Oxygen Activity in Lithium-Rich Layered Oxides. Nat. Commun. 2018, 9, 947 10.1038/s41467-018-03403-9. 29507369 PMC5838240

[ref50] Chen D.; Kan W. H.; Chen G. Understanding Performance Degradation in Cation Disordered Rock-Salt Oxide Cathodes. Adv. Energy Mater. 2019, 9, 1901255 10.1002/aenm.201901255.

[ref51] Rahman M. M.; Lin F. Oxygen Redox Chemistry in Rechargeable Li-Ion and Na-Ion Batteries. Matter 2021, 4, 490–527. 10.1016/j.matt.2020.12.004.

