# Supplementary material for: Fluorination Effect on Lithium- and Manganese-Rich Layered Oxide Cathodes
Source: ACS Energy Lett. 2024 Feb 27;9(3):1249–60. doi: 10.1021/acsenergylett.3c02697 (PMC10928714; doi:10.1021/acsenergylett.3c02697)
Supplement: Supplementary file 1 — nz3c02697_si_001.pdf [file nz3c02697_si_001.pdf]

Supporting Information

Fluorination Effect on Lithium- and Manganese-Rich Layered Oxide Cathodes

Faxing Wang, [1] Peng Zuo, [2] Zhichen Xue, [3] Yijin Liu, [3] Chongmin Wang [2] and Guoying Chen [1,*]

[1] *Energy Storage and Distributed Resources Division, Lawrence Berkeley National Laboratory, FBerkeley, California 94720, United States*

[2] *Environmental Molecular Sciences Laboratory, Pacific Northwest National Laboratory, Richland, WA 99354, United States*

[3] *Stanford Synchrotron Radiation Lightsource, SLAC National Accelerator Laboratory, Menlo Park, California 94025, United States*

Experimental method

Synthesis

LNMO and F-LNMO crystals were prepared by a modified molten-salt method. [1, 2] Stoichiometric amounts of Li_2CO_3, $Ni(CH_3COO)_2·4H_2O$ and $Mn(CH_3COO)_2·4H_2O$ (Sigma-Aldrich) were used as lithium, nickel and manganese precursors, respectively. Specifically, 3.9 mmol Li_2CO_3 (30% excess), 1 mmol $Ni(CH_3COO)_2·4H_2O$ and 3 mmol $Mn(CH_3COO)_2·4H_2O$, were mixed with various amounts of KF/LiF (mole ratio 49:51) through ball milling. KCl was used as the molten salt for the reactions. The mole ratio between the transition-metal salts and the KCl flux was kept at 8. The amounts of the fluoride salts were set to be 0, 0.05, 0.125 and 0.25 mmol for LNMO, LNMO-F1, LNMO-F2.5 and LNMO-F5, respectively. The mixed powders were annealed at 450 °C for 6 h and further heated at 900 °C for 12 h. The heating rate was 10 °C min^{-1}. After calcination, the furnace was cooled down to room temperature naturally. The obtained samples were washed with deionized water to remove soluble salts by centrifugation and then filtered, and finally dried at 100 °C in an oven for 12 h.

Electrochemical measurements

The composite cathodes were prepared by mixing the as-prepared LNMO or F-LNMO sample, a polyvinylidene fluoride (PVDF) binder and a conductive carbon black (mass ratio 8:1:1) in an N-methyl-2-pyrrolidone (NMP) solvent. The resulting slurry was then cast on an aluminum foil and dried at 100 °C for 12 h in a vacuum oven. Electrode discs with a size of 1.6 cm^2 and an average active mass loading of ~3 mg cm^{-2} were cut out and used as the working electrodes in cell testing. The 2032-type coin cells were assembled in an argon-filled glovebox, using Li foil (Alfa-

Aesar) as both counter and reference electrodes. Celgard 2400 membrane and 1.2 M $LiPF_6$ in EC/EMC (volume ratio 3:7) were used as the separator and electrolyte, respectively. The assembled half-cells were galvanostatically cycled using a VMP3 multichannel potentiostat/galvanostat. All electrochemical measurements were performed at room temperature with a current density of 20 mA g^{-1}.

Characterization

Morphologies were evaluated by using an scanning electron microscope (JEOL JSM-7500F field emission) at a 10 kV accelerating voltage. The crystallinity and phase purity of LNMO and F-LNMO samples were analyzed by using synchrotron X-ray diffraction patterns collected at beam line 11-3 (λ = 1.54 Å) at the Stanford Synchrotron Radiation Lightsource (SSRL) of the SLAC National Accelerator Laboratory. LaB_6 crystals were used for calibration. Soft X-ray absorption spectroscopy analysis for the powder samples and cycled electrodes was conducted at SSRL beam line 10-1 and 8-2, using a 1000 l mm^{-1} spherical grating monochromator with 20 mm entrance/exit slits, a 0.2 eV energy resolution, and a 1 mm^2 beam spot. The XPS measurements were performed using the K-Alpha XPS System from Thermo Scientific. The photon source was a monochromatized Al K α line ($h\nu$ = 1486.6 eV). The spectra were acquired using a spot size of 400 μm and constant pass energy. The spectra were obtained using a combined low energy electron/ion flood source for charge neutralization and a dual monoatomic and gas cluster argon ion source for depth profiling and sample cleaning. The STEM/EELS data were collected using JEOL JEM-ARM200CF microscope (200 kV) with a probe spherical aberration corrector, Gantan Quantum EELS system and a JEOL SDD-detector with a 100 mm^2 X-ray sensor at Pacific Northwest National Laboratory. The inner and outer collection angles of annular dark field

detector were set at 68 and 280 mrad for STEM-HAADF/BAF imaging. FIB was used to section single-crystal particles before analysis which were mapped using STEM to analyze the surface facets. The atomic structures were captured along the [010] direction of the layered structure. Operando DEMS measurements were performed in a custom-made Swagelok cell in Ar-filled glovebox as described in our previous publication. [3] The Swagelok cells were assembled with a LNMO or LNMO-F5 cathode and a Li metal anode in 80 μl of 1 M $LiPF_6$ in 3:7 v/v of EC:DEC electrolyte. The assembled Swagelok cells were cycled at a current density of 20 mA g^{-1} between 2 and 4.8 V using a Bio-Logic VSP-series potentiostat under a positive Ar pressure (~ 1.2 bar). The gas evolution was monitored *in situ* using the custom-built DEMS apparatus, using a previously described procedure. [4]

Supplementary figures

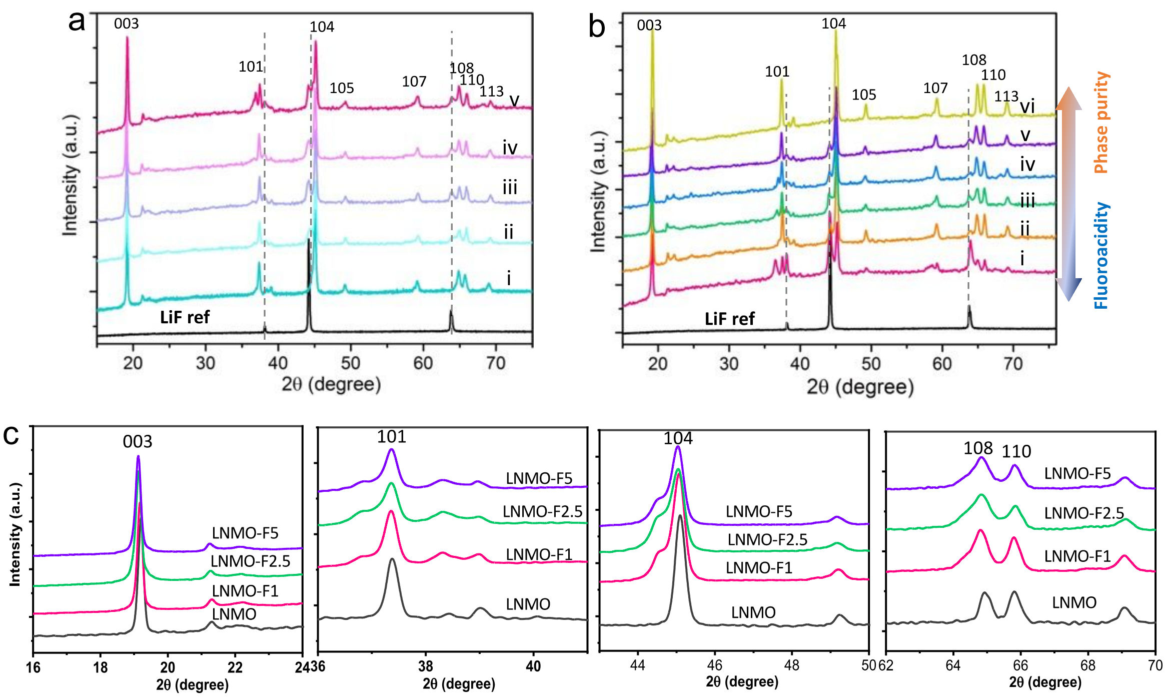

Figure S1. a) XRD patterns of LNMO and F-LNMO samples synthesized with LiF. i: LNMO, ii: LNMO-F1, iii: LNMO-F2.5, iv: LNMO-F5, and v: LNMO-F10), b) XRD patterns of LNMO-F2.5 samples synthesized with different fluoride salts. i: LiF–CaF_2, ii: LiF, iii: LiF–NaF, iv: NaF–CaF_2, v: NaF–MgF_2 and vi: LiF–KF. c) The expanded views of the characteristic (003), (101), (104), and (108)/(110) peaks from laboratory powder XRD patterns (shown in Figure 1a) collected on the as-synthesized crystal samples.

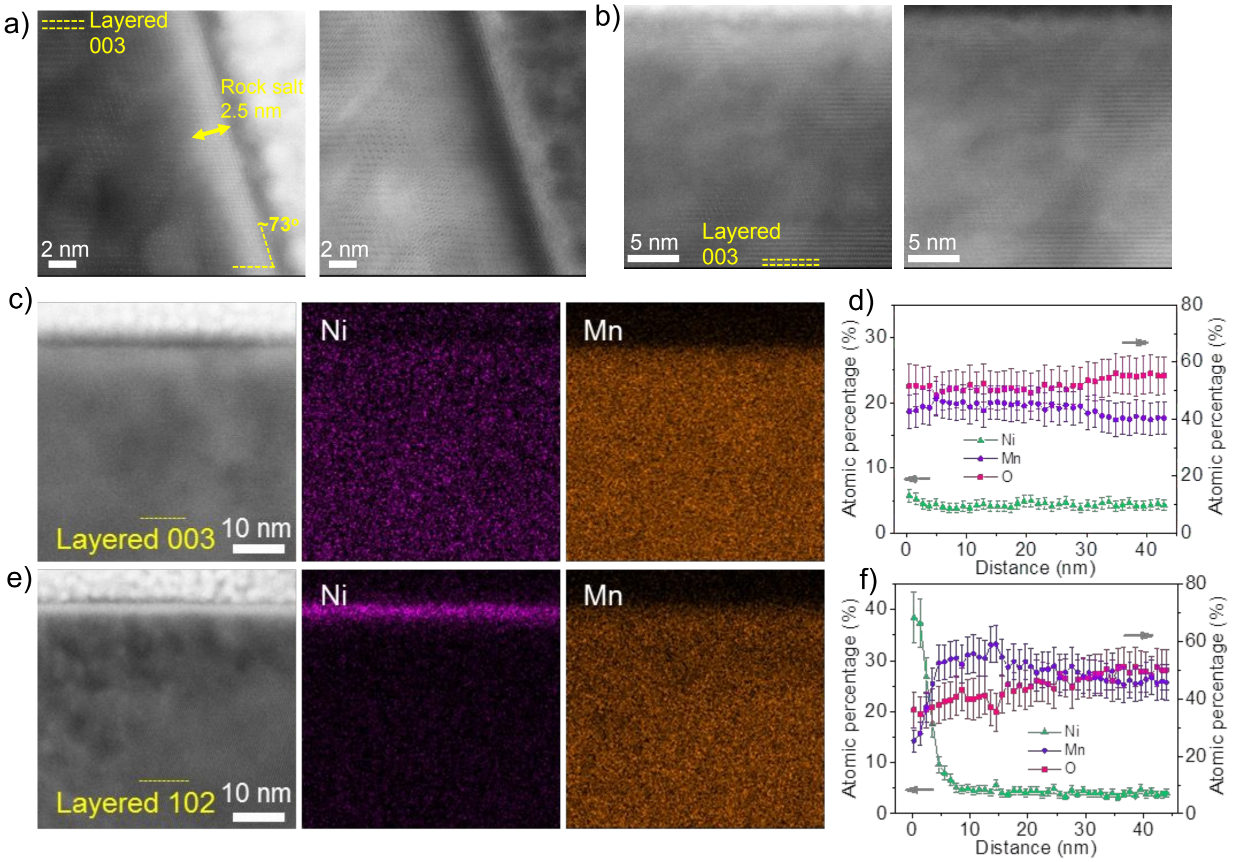

Figure S2. STEM-HAADF (left) and STEM-ABF (right) images of LNMO along: a) [010] and b) $[10\bar{1}]$ zone axes. STEM-EDX mapping and the estimated atomic percentages of the indicated elements at c, d) (102) and e, f) (003) crystal facets of LNMO, respectively.

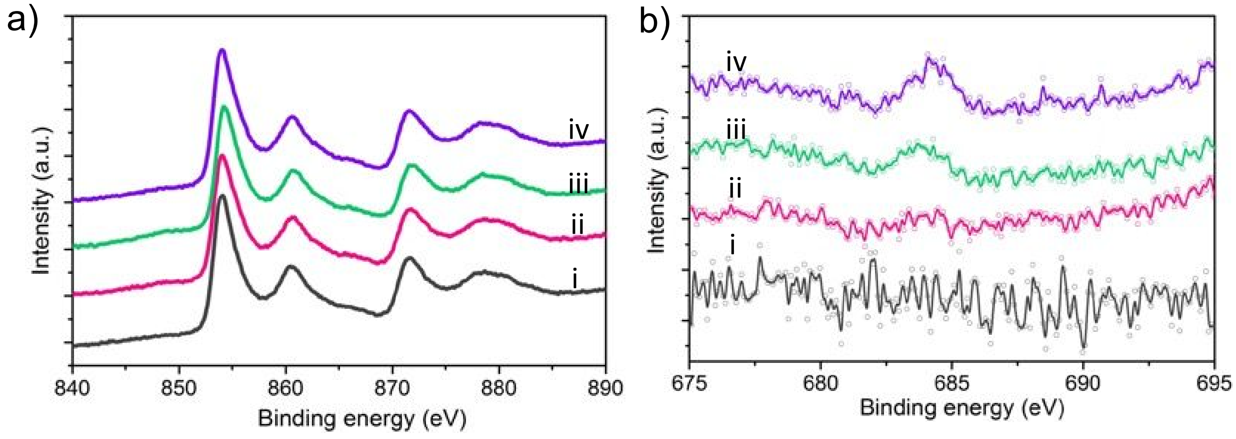

Figure S3. a) Ni 2*p* and b) F 1*s* XPS spectra of the pristine crystal samples. Samples are labeled as i: LNMO, ii: LNMO-F1, iii: LNMO-F2.5 and iv: LNMO-F5.

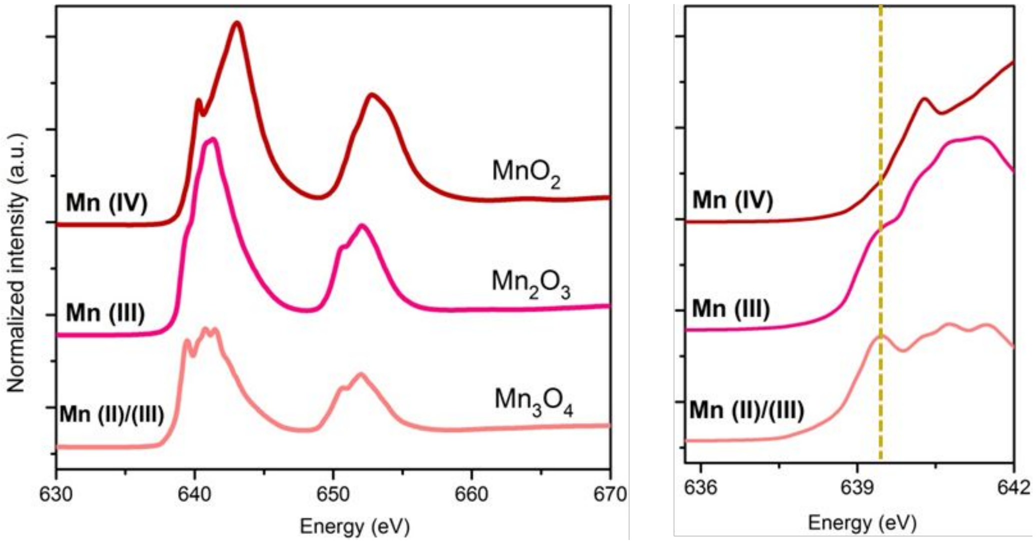

Figure S4. Mn *L*-edges sXAS profiles of MnO_2, Mn_2O_3 and Mn_3O_4 references.

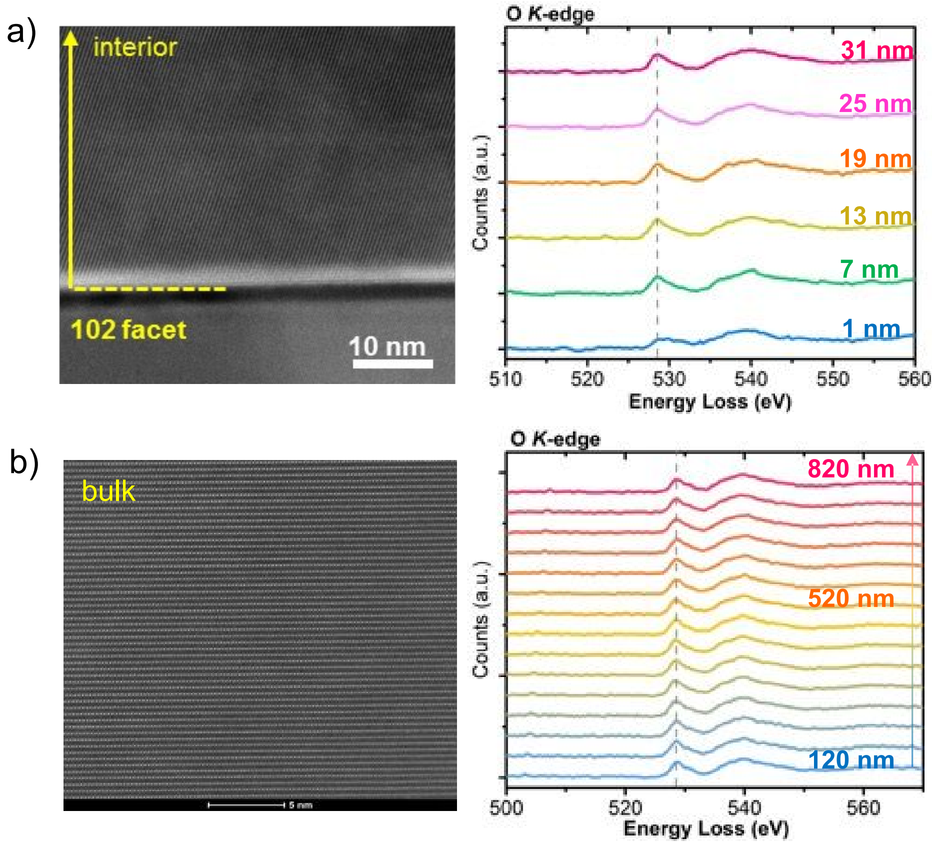

Figure S5. STEM-HAADF (left) and O *K*-edge STEM-EELS spectra (right) collected on a LNMO-F5 crystal: a) in the (102) surface region of 1 to 31 nm at a step size of 6 nm and b) in the bulk region from 120 to 820 nm at a step size of 50 nm.

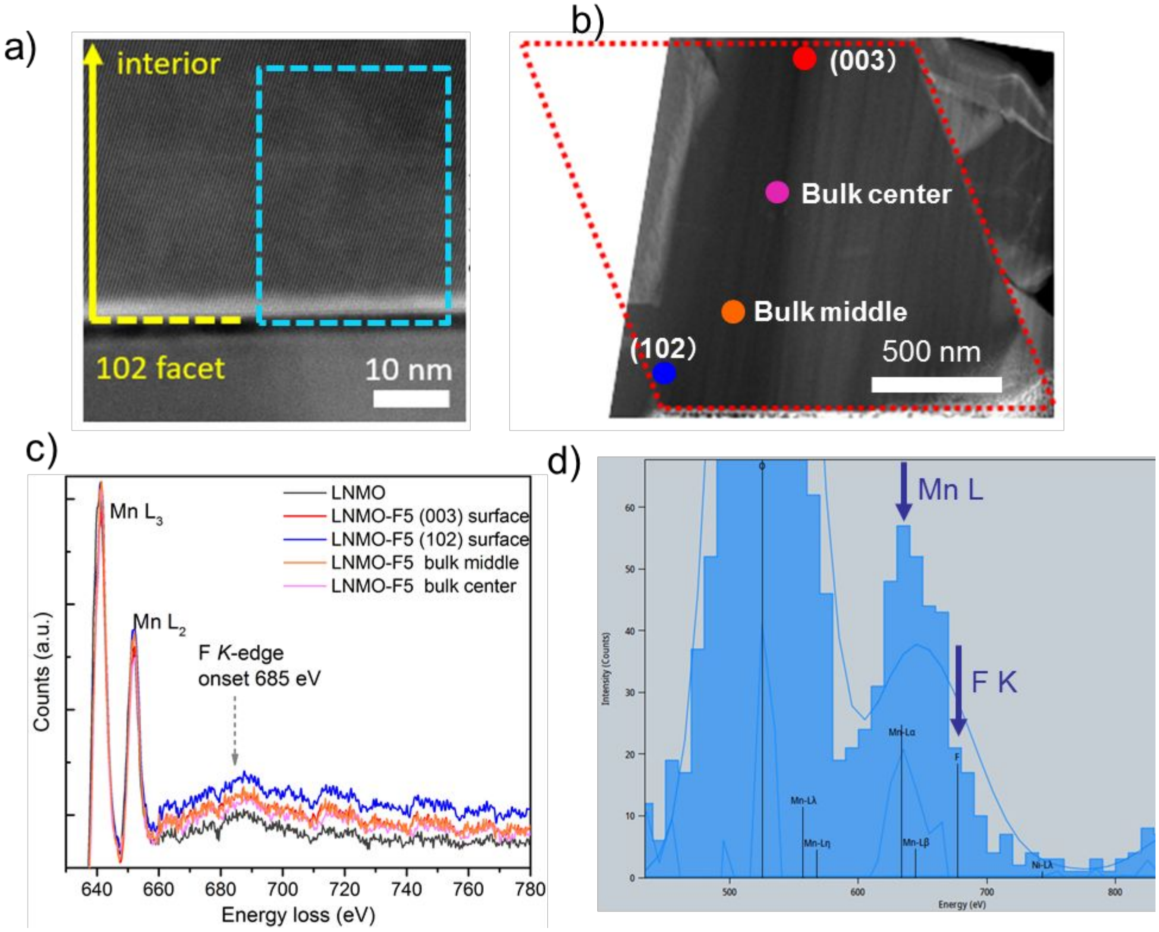

Figure S6. a) STEM-HAADF image of LNMO-F5 at (102) surface and b) pixel-by-pixel point acquisition at (102)/(003) surfaces and bulk of LNMO-F5 for the EELS spectra. c) The corresponding Mn *L*-edge EELS profiles of LNMO and LNMO-F5 collected at the different points in c). d) Overlapping of F *K*-edge and Mn *L*-edge in STEM-EDX analysis.

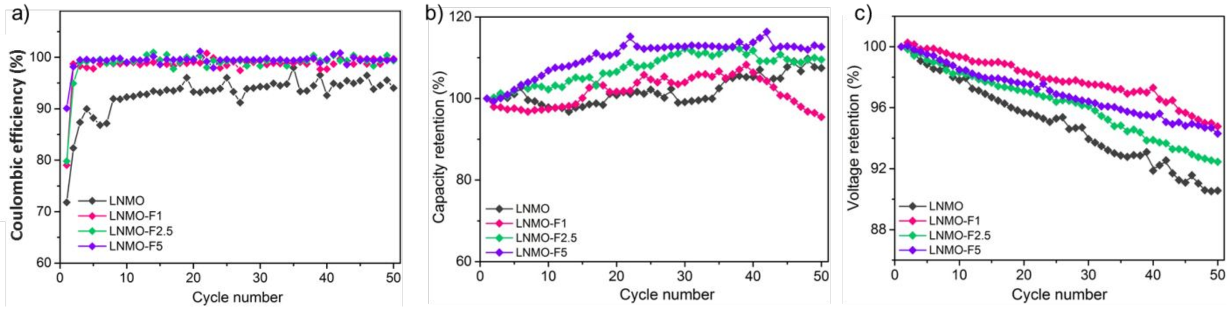

Figure S7. a) Coulombic efficiencies, b) specific capacity retention and c) energy density retention of LNMO and F-LNMO cathodes cycled at 0.1C in the voltage range of 2 – 4.8 V.

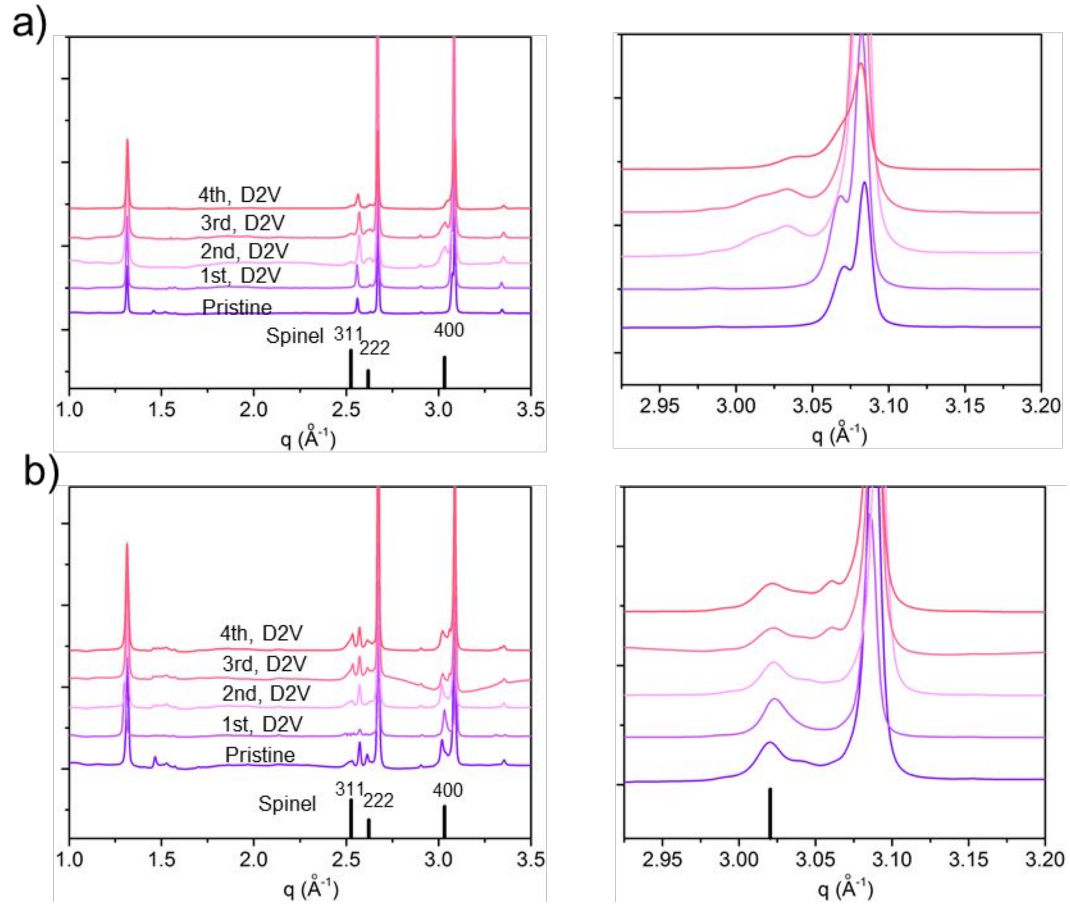

Figure S8. Synchrotron XRD patterns of pristine and recovered electrodes after various cycles: a) LNMO and b) LNMO-F5. Right panels show the expanded views.

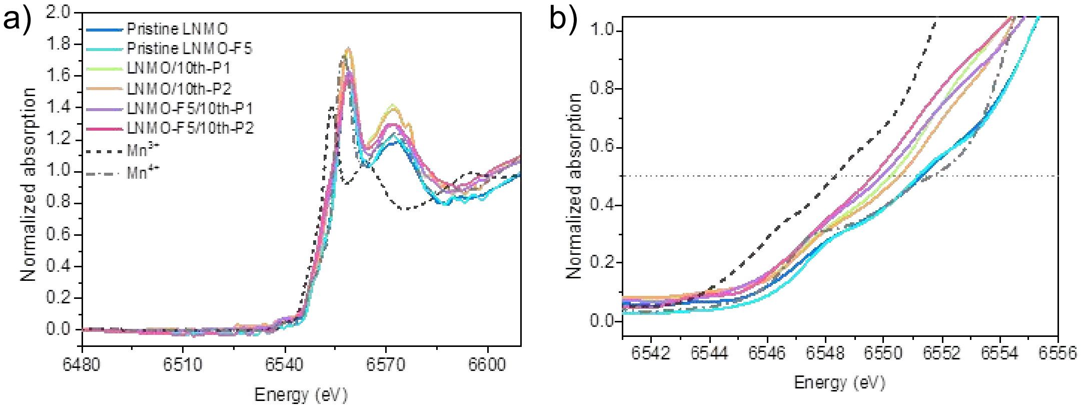

Figure S9. The Mn *K*-edge XANES spectra of pristine LNMO and LNMO-F5 collected at various spots from the TXM images in Figure 6.

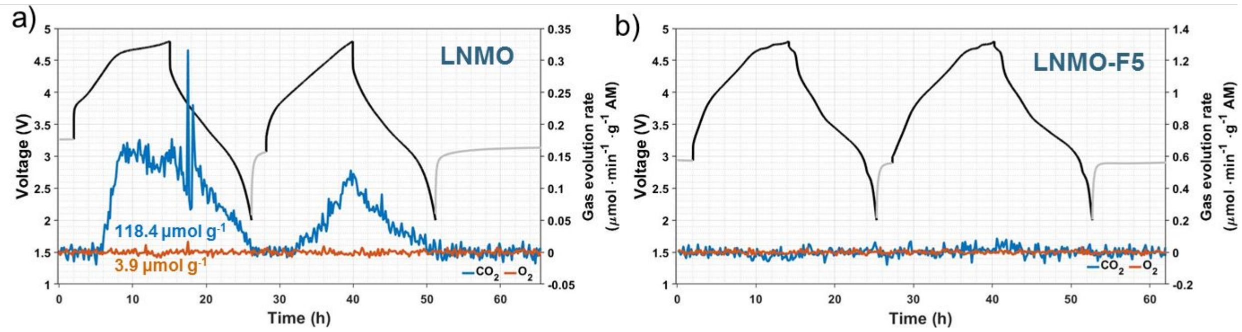

Figure S10. Operando DEMS analysis of a) LNMO and b) LNMO-F5 with voltage profiles and the corresponding O_2/CO_2 gas evolution during the initial 2 cycles.

Table S1. Properties of F-containing salts used for *in situ* fluorination of LNMO crystals.

F-salts	LiF–KF	NaF–MgF_2	NaF–CaF_2	LiF–NaF	LiF	LiF–CaF_2
Mole ratio (%)	51:49	78:22	69:31	60:40	100	80:20
Melting point (^oC)	492	835	814	652	848	760

Table S2. Chemical compositions of pristine LNMO and F-LNMO samples determined by ICP analysis.

**Samples**	**Target composition**	**Metal content (ICP)**		
		**Li**	**Ni**	**Mn**
LNMO	$Li_{1.2}Ni_{0.2}Mn_{0.6}O_2$	1.204	0.189	0.611
LNMO-F1	$Li_{1.2}Ni_{0.2}Mn_{0.6}O_{1.99}F_{0.01}$	1.192	0.193	0.607
LNMO-F2.5	$Li_{1.2}Ni_{0.2}Mn_{0.6}O_{1.975}F_{0.025}$	1.220	0.211	0.588
LNMO-F5	$Li_{1.2}Ni_{0.2}Mn_{0.6}O_{1.95}F_{0.05}$	1.221	0.216	0.584

Supplementary references

1. K. Luo, M. R. Roberts, R. Hao, N. Guerrini, E. Liberti, C. S. Allen, A. I. Kirkland, P. G. Bruce. One-Pot Synthesis of Lithium-Rich Cathode Material with Hierarchical Morphology. *Nano Lett.* **2016**, *16*, 7503−7508.

2. X. Li, Y. Qiao, S. Guo, Z. Xu, H. Zhu, X. Zhang, Y. Yuan, P. He, M. Ishida, H. Zhou. Direct Visualization of the Reversible O_2^-/O^- Redox Process in Li-Rich Cathode Materials. *Adv. Mater.* **2018**, *30*, 1705197.

3. J. Sun, C. Sheng, X. Cao, P. Wang, P. He, H. Yang, Z. Chang, X. Yue, H. Zhou. Restraining Oxygen Release and Suppressing Structure Distortion in Single-Crystal Li-Rich Layered Cathode Materials. *Adv. Funct. Mater.* **2022**, *32*, 2110295.

4. J. Ahn, Y. Ha, R. Satish, R. Giovine, L. Li, J. Liu, C. Wang, R. J. Clement, R. Kostecki, W. Yang, G. Chen. Exceptional Cycling Performance Enabled by Local Structural Rearrangements in Disordered Rocksalt Cathodes. *Adv. Energy Mater.* **2022**, *12*, 2200426.
